# Protein Conformational Space at the Edge of Allostery: Turning a Nonallosteric Malate Dehydrogenase into an “Allosterized” Enzyme Using Evolution-Guided Punctual Mutations

**DOI:** 10.1093/molbev/msac186

**Published:** 2022-09-03

**Authors:** Antonio Iorio, Céline Brochier-Armanet, Caroline Mas, Fabio Sterpone, Dominique Madern

**Affiliations:** Laboratoire de Biochimie Théorique, CNRS, Université de Paris, UPR 9080, Paris, France; Institut de Biologie Physico-Chimique-Fondation Edmond de Rothschild, PSL Research University, Paris, France; Laboratoire de Biométrie et Biologie Évolutive, Univ Lyon, Université Lyon 1, CNRS, UMR5558, Villeurbanne, France; CEA, CNRS, IBS, Univ. Grenoble Alpes, Grenoble, France; Laboratoire de Biochimie Théorique, CNRS, Université de Paris, UPR 9080, Paris, France; Institut de Biologie Physico-Chimique-Fondation Edmond de Rothschild, PSL Research University, Paris, France; CEA, CNRS, IBS, Univ. Grenoble Alpes, Grenoble, France

**Keywords:** evolution of allostery, Molecular dynamics and evolvability, Lactate and malate dehydrogenase, Hidden conformational states of enzymes

## Abstract

We unveil the intimate relationship between protein dynamics and allostery by following the trajectories of model proteins in their conformational and sequence spaces. Starting from a nonallosteric hyperthermophilic malate dehydrogenase, we have tracked the role of protein dynamics in the evolution of the allosteric capacity. Based on a large phylogenetic analysis of the malate (MalDH) and lactate dehydrogenase (LDH) superfamily, we identified two amino acid positions that could have had a major role for the emergence of allostery in LDHs, which we targeted for investigation by site-directed mutagenesis. Wild-type MalDH and the single and double mutants were tested with respect to their substrate recognition profiles. The double mutant displayed a sigmoid-shaped profile typical of homotropic activation in LDH. By using molecular dynamics simulations, we showed that the mutations induce a drastic change in the protein sampling of its conformational landscape, making transiently T-like (inactive) conformers, typical of allosteric LDHs, accessible. Our data fit well with the seminal key concept linking protein dynamics and evolvability. We showed that the selection of a new phenotype can be achieved by a few key dynamics-enhancing mutations causing the enrichment of low-populated conformational substates.

## Introduction

Allostery is the process in which protein catalytic efficiency is regulated by the binding of an effector at a specific distal site from the catalytic site (Wodak et al. 2019). According to initial models of allostery, the process relies on a ligand-dependent conformational change between a tense inactive state (T-state) and a relaxed active state (R-state) (Monod et al. 1965; Koshland et al. 1966). Later, extensive analyses have shown that allosteric regulation is grounded in the protein dynamics, leading to the unifying and general “ensemble model” of allostery (Hisler et al. 2012; Motlagh et al. 2014; Biddle et al. 2021). In this model, the allosteric capacity of an enzyme depends on the reorganization of the protein conformational landscape, induced by events such as interactions with other protein partners or ligands, local unfolding, and physicochemical variations of the environment (Schrank et al. 2009; Arai et al. 2010; Lisi et al. 2018; Halgand et al. 2020; Iorio et al. 2021).

Protein superfamilies encompassing allosteric and nonallosteric enzymes are under study to disclose the molecular mechanisms of allosteric regulation (Huang et al. 2014; Suplatov and Švedas 2015). Indeed, deciphering the evolutionary history of these super families can enhance our understanding of the structure, function, and dynamical relationships that allowed allostery to emerge (reviewed in Modi et al. 2021). Here, we focus on the lactate/malate dehydrogenase (LDH/MalDH) superfamily. LDH (EC 1.1.1.27) are 2-ketoacid: NAD(P)-dependent dehydrogenases that catalyze the reversible conversion of 2-hydroxyacids to the corresponding 2-ketoacids. LDHs are involved in energy metabolism and operate at the final stage of glycolysis (Holbrook et al. 1975; Fersht 1985; Adeva-Andany et al. 2014). They reversibly transform pyruvate (PYR) into lactate using NADH as coenzyme. Most bacterial LDHs are allosterically regulated, that is, they display sigmoid kinetics as a function of PYR concentration (homotropic activation) and are activated in the presence of the allosteric effector fructose 1,6-bisphosphate (FBP) (heterotropic activation) (Garvie 1980; Schroeder et al. 1988; Arai et al. 2002; Feldman-Salit et al. 2013; Taguchi 2017). In contrast, LDHs from eukaryotes are thought as nonallosteric, a consideration mainly based on the characterization of vertebrates enzymes, even if this has been recently challenged (Iacovino et al. 2022).

The catalytic mechanism of LDH has been studied extensively. In its competent catalytic state, LDH catalyzes the direct transfer of a hydride ion from the pro-R face of NADH to the C2 carbon of PYR to produce lactate. The complete process involves a set of several amino acids, with specific roles (Burgner and Ray 1984; Clarke et al. 1986, 1988; Deng et al. 1994, 2011; McClendon et al. 2005). The amino acids, listed according to the LDH nomenclature (Eventoff et al. 1977), are: the reactive histidine H195, glutamic acid D168 that helps to polarize H195, arginine R109 that stabilizes the transition state, the substrate-binding residue arginine R171, and glutamine Q102 that sustains specifically the recognition of the PYR. In the catalytic site, threonine T246, which is conserved in all LDH sequences, also contributes to the correct geometry and favorable chemistry, ensuring efficient catalysis (Bur et al. 1989; Sakowicz et al. 1993). In LDHs, there is always an acid residue (D or E) at position 199, which contributes to charge neutrality when the mobile loop covers the catalytic site (Wilks et al. 1988).

The crystal structures of allosteric LDHs without (Apo) and with ligands (Holo) revealed large conformational changes that are representative of the transition between the T- and R-states (Piontek et al. 1990; Wigley et al. 1992; Iwata et al. 1994; Auerbach et al. 1998; Winter et al. 2003; Coquelle et al. 2007; Arai et al. 2010; Colletier et al. 2012; Matoba et al. 2014; Kolappan et al. 2015). These structural reorganizations involve changes such as the expansion or compaction of the quaternary scaffold together with some helix sliding and local rearrangements within the catalytic site (see the review by Taguchi 2017). In structures typical of the R-active state, a mobile loop that carries the substrate discriminating glutamine Q102 covers the catalytic site, allowing its dehydration and the correct anchoring of PYR with the side chain of R171, which protrudes within the catalytic site. In contrast, representative structures of the T-inactive state show that the R171 side chain is outside the catalytic site, demonstrating that its position is a strong proxy of allosteric capacity (Colletier et al. 2012; Iorio et al. 2021). Furthermore, in allosteric LDHs, H68 and R171 are close to each other with coordinated side-chain conformations. In Apo structures of LDHs, H68 is in a conformation that hinders the entry of the side chain of R171 into the catalytic site.

In addition to LDHs, the LDH/MalDH superfamily includes also malate dehydrogenases (MalDH, EC 1.1.1.37) and hydroxyisocaproate dehydrogenases (HicDH) (Madern 2002; Madern et al. 2004; Boucher et al. 2014). MalDHs are involved in the tricarboxylic acid cycle and oxidize oxaloacetate (OAA) into malate using NAD(P)Has coenzyme (Minárik et al. 2002). Despite a similar catalytic mechanism and common structural scaffold, the substrate recognition mechanism between LDHs and MalDHs is very stringent. This discriminating effect relies mainly on the nature of the amino acid at position 102, which is Q102 in LDHs and R102 in MalDHs (Wilks et al. 1988; Chapman et al. 1999). MalDHs have been divided into three subgroups: the dimeric MalDH type 1 and MalDH type 2 and the tetrameric MalDH type 3. Phylogenetic analyses showed that at least four MalDH toward LDH functionality occurred. More precisely, canonical LDH, LDH of *Plasmodium*- and *Cryptosporidium*-related species derived independently from ancient MalDH type 3, while the LDH of *Trichomonas vaginalis* derived from MalDH type 1 (Madern 2002; Zhu and Keithly 2002; Madern et al. 2004; Boucher et al. 2014; Steindel et al 2016; Brochier-Armanet and Madern 2021). Regarding canonical LDH, the transition occurred from within an intermediate group of sequences with various affinities and substrate discrimination capacities, which can be considered as a reservoir of enzymes prone to evolve new phenotypes (Brochier-Armanet and Madern 2021). Sequences of this intermediate group branch in-between MalDH type 3 and LDH. They form several subgroups (referred as to A–P) and harbor variable combinations of amino acids that are usually specific of either LDH (Q102, D/E199, and T246) or MalDH type 3 (R102, M199, and A/S246). The LDH capacity to recognize and use PYR as substrate was in part the consequence of the acquisition of a permissive amino acid, a threonine (T) at amino acid position 246, early during the diversification of the intermediate group of MalDH (Brochier-Armanet and Madern 2021). T246 is also considered unfavorable for OAA binding (Bur et al. 1989). Strengthening this proposal, some MalDHs of the intermediate group that harbors T246 recognize both OAA and PYR as substrates (Roche et al. 2019). Later, the fixation of a glutamine (Q) in position 102 granted binding specificity toward PYR (Brochier-Armanet and Madern 2021). This substitution occurred in the stem of the canonical LDH clade.

Studying enzymes from the intermediate group could provide useful information to describe how allosteric properties evolved within the MalDH/LDH super family. Until now, no allosteric behavior has been reported for MalDHs type 3, whereas some MalDHs from the intermediate groups of sequences displayed a latent (i.e., hidden) allosteric capacity, underpinning their close relationship with LDHs (Roche et al. 2019; Katava et al. 2020; Brochier-Armanet and Madern 2021). Note that the complete allosteric regulation via homotropic and heterotropic activation, as in the case of canonical allosteric LDHs, has not been reported in any enzyme of the MalDH intermediate group.

In this study, by comparing structures, we identified a combination of three critical amino acids (at positions 68, 102, and 250) that could be related to the emergence of allosteric capacity from MalDHs to LDHs and we showed that the nature of amino acid at position 250 plays a major role for the selection of allosteric capacity in LDH. This hypothesis was then addressed by using site-directed mutagenesis on the enzyme of *Archaeoglobus fulgidus*, a dimeric MalDH belonging to the intermediate group that has been characterized biochemically and structurally (Madern et al. 2001; Irimia et al. 2004). The replacement of P250 by an isoleucine in conjunction with the replacement of the R102 by Q102 revealed important changes in dynamical properties that allowed the nonallosteric *A. fulgidus* MalDH to turn into a homotropically activated dimeric LDH. Our study strongly suggests that the allosteric properties in LDHs emerged from nonallosteric MalDHs, via an intermediate group of enzymes, in which a few key mutations induced a reorganization of their conformational landscape rendering them prone to evolve allosteric regulation.

## Results

### Investigating the Phylogeny of the LDH/MalDH Superfamily Suggests That the Universal Conserved Position I250 Could Be a Key Determinant for Allostery

**Fig. 1.**
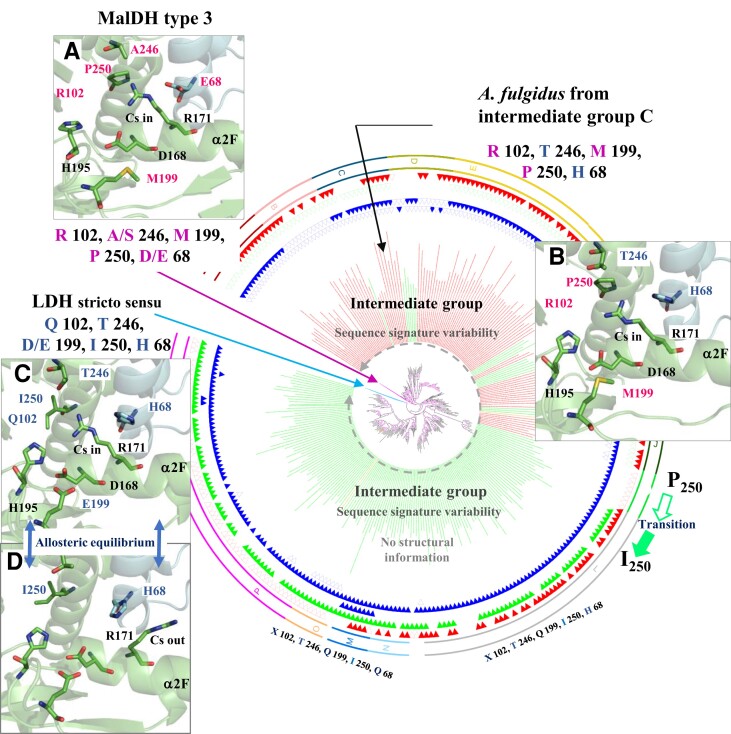
Unrooted maximum likelihood phylogeny of the LDHs, MalDH type 3, and intermediate MDHs. An extended view of the tree is presented in supplementary figure S1, Supplementary Material online. The blue and purple branches correspond to LDHs and MalDH type 3 (for clarity, the corresponding clades have been collapsed and are indicated by arrows). Branches of the intermediate group of MalDH are colored according to the taxonomy: green indicates bacterial sequences, red archaeal sequences, and yellow eukaryotic sequences. Sequences of the MalDH intermediate group display mixed combinations of the LDH and MalDH type signatures. The circular regions made of triangles correspond to critical residues: empty triangles correspond to residues usually found in MalDH type 3, while filled residues are similar to those found in LDHs. More precisely, the most inner circle corresponds to Q102 (filled blue triangles), R102 (empty triangles), or other residues (no triangle), the second circle to T246 (filled blue triangles), A/S246 (empty blue triangles), or other residues (no triangle), the filled triangles on the third circle design an acidic residue (D/E199) that ensure charge neutrality within the catalytic site of LDH, the fourth circle to I250 (filled green triangles), P250 (empty green triangles), or other residues (no triangle), and the fifth circle to H68 (filled red triangles), D/Q68 (empty triangles), or other residues (no triangle).The *A*, *B*, and *C* panels show the structure of the catalytic sites (close-up views). (*A*) MalDH type 3 from *Chloroflexus aurantiacus* (Bacteria) (PDB ID. 4CL3), (*B*) Intermediate MalDH (subgroup C) from *Archaeoglobus fulgidus* (Archaea) (PDB ID. 2XOI). (*C* and *D*) LDH from *Thermus thermophilus* (Bacteria) (PDB ID. 2V7*P*, 2V6*M* being Holo and Apo state, respectively). Residues colored in pink and blue are typical of MalDHs and LDHs, respectively. For the sake of clarity, the helix α1*G*/α2*G* is not shown. The nature of the amino acid at position 102 for each enzyme is indicated, but due to the location at a large distance, it is not represented. Amino acids are numbered according to the normalized nomenclature of Eventoff et al. (1977). The correspondence between the primary sequence and the normalized numbering for LDH is shown in supplementary table S1, Supplementary Material online. Cs, conformational substate.

We used the recently published LDH/MalDH phylogeny (Brochier-Armanet and Madern 2021) to reveal candidate amino acids that could be linked to allosteric behavior. More precisely, we focused on the large clade encompassing the LDHs, the MalDH type 3, and the intermediate group that branches in-between LDHs and MalDH type 3. The intermediate group displayed enzymes with mixed properties with respect to substrate recognition (Brochier-Armanet and Madern 2021). Based on structural and sequence comparisons, we explored the organization of the catalytic site of LDH, MalDH type 3, and the intermediate group of MalDH (fig. 1*A–D*).

The *Thermus thermophilus* LDH enzyme harbors the canonical Q102, D/E199, T246 amino acid signature, the *Chloroflexus aurantiacus* MalDH type 3 contains the typical R102, M199, A/S246 amino acids, while the catalytic site of the *A. fulgidus* MalDH of the intermediate group displays a mix signature: R102, M199, and T246 (fig. 1 and supplementary fig. S1, Supplementary Material online). In allosteric LDHs, H68 and R171 displayed synchronized lateral side-chain reorganizations, with H68 mimicking the role of a “door” that controls the localization of R171 within conformational substates (Cs-in) or outside (Cs-out) the catalytic site (fig. 1*C*and*D*). In nonallosteric MalDH 3, there is a D at position 68. Sequences from the intermediate groups harbor either D68 or H68 (red triangles, fig. 1 and supplementary fig. S2, Supplementary Material online), suggesting that the D68 to H transition occurred in this group prior to being fixed in LDH.

The structural analysis (fig. 1) showed that the amino acid at position 250 is located in close vicinity to R171 and the amino acid at position 246, in agreement with previous reports indicating it is close to the reactive extremity of NADH (Dalhus et al. 2002; Irimia et al. 2004; Coquelle et al. 2007). Interestingly, the amino acid at position 250 was also proposed to distinguish between LDHs (I250) and MalDH type 3 (P250) (Madern 2002). Yet, the role of the amino acid at this position has never been experimentally analyzed. In light of these considerations, we investigated the evolution of residues at positions 68, 102, and 250 along the phylogeny of LDHs, MalDH type 3, and intermediate MalDHs (fig. 1 and supplementary fig. S2, Supplementary Material online).

The mapping disclosed a strong association between R102, P250, and D/Q68 in MalDH type 3, and between Q102, I250, andH68in LDHs (supplementary fig. S1, Supplementary Material online), suggesting these amino acid signatures may be linked to specific enzymatic properties, such as allosteric capacity and substrate recognition. Regarding the intermediate group of MalDH, mixed combinations of LDHs and MalDH type 3 signatures are observed (fig. 1 and supplementary fig. S2, Supplementary Material online). More precisely, most sequences harbor: 1) R102, P250, as MalDH type 3, associated with the H68 observed in LDHs, 2) I250 and H68, as LDHs, but neither Q102 nor R102, or 3) I250 as LDHs, D68 as MalDH type 3, but neither Q102 nor R102. While most sequences from the intermediate group display A/S246 like MalDH type 3, some of them harbor H68, R102, and P250, carries also T246-like LDH sequences (fig. 1 and supplementary fig. S2, Supplementary Material online). They provide, therefore, an opportunity to examine the most favorable combination, using a minimal set of evolutionary-related mutations, which may turn a nonallosteric MalDH into an allosterized enzyme. The crystal structures of only two MalDH enzymes from the intermediate group that display the H68, R102, T246, and P250 signature sequence are deposited at PDB: 2XOI and 6QSS from the archaea *A. fulgidus* and *Ignicoccus islandicus*, respectively (Irimia et al. 2004; Roche et al. 2019). Each display peculiar discrepancies with respect to the canonical tetrameric association that prevails in the LDH/MalDH super family. The *A. fulgidus* enzyme is a dimer, likely because of a six amino acid deletion at the interface that participates to tetrameric assembly (Madern et al. 2001; Irimia et al. 2004), while the *I.isl* enzyme is a “twisted” tetramer with a major displacement, at the dimer of dimers that make the final assembly (Roche et al. 2019).

To go further, we investigated the role of amino acids at positions 102 and 250 using the enzyme from *A. fulgidus* as template. More precisely, three mutants were designed. The first one, mutant 1, contains the single R to Q mutation at position 102 (R102Q) that is intended to modify the substrate recognition, from OAA toward PYR making this enzyme an LDH, while the proline in position 250 is unchanged. The second one, mutant 2, allows us to test the effect of the substitution of proline by isoleucine at position 250 (P250I), while the R102 is not modified in order to keep the MalDH functionality unaltered. The third and final one, mutant 3 (R102Q and P250I), combines both mutations.

### Effect of Mutations on Protein Stability and Activity Profiles

We first checked that the mutations were without any major effects on the quaternary assembly, fold, and stability of the resulting purified enzymes. The Wt *A. fulgidus* MalDH and the three mutants were eluted on a size exclusion chromatography (SEC) column with a similar profile. As expected for dimeric enzymes, they displayed a similar elution volume (Ve = 14.5 ml) higher than the value of 13.5 ml corresponding to a tetrameric LDH used as control (supplementary fig. S3, Supplementary Material online).

**Fig. 2.**
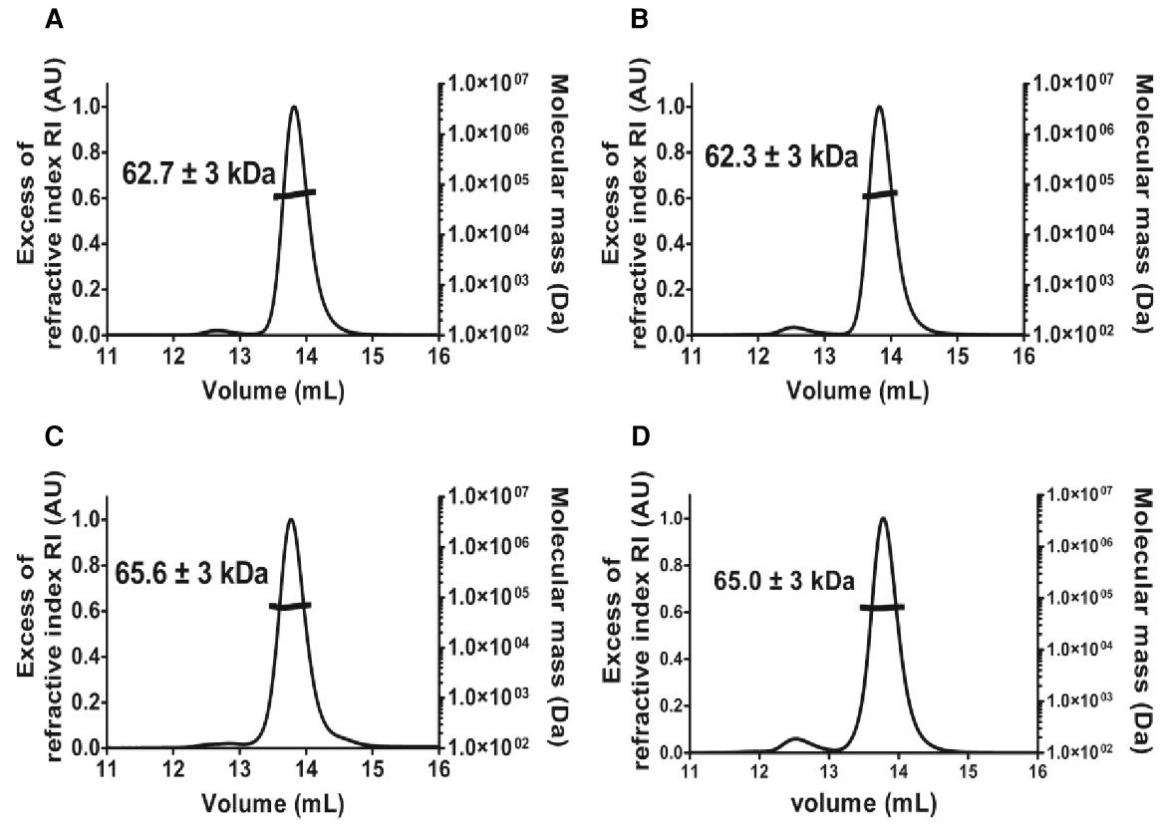
Oligomeric state determination of the *A. fulgidus* MalDH and mutants using SEC–MALLS analysis. The chromatogram shows the elution profile monitored by excess refractive index (left ordinate axis) and the molecular weight as dashed line (right ordinate axis) derived from MALLS and RI measurements. The estimated average molecular weight is indicated on the graph. (*A*) Wt *A. fulgidus* MalDH, (*B*) mutant 1, (*C*) mutant 2, and (*D*) mutant 3.

We then monitored their apparent molecular weight using a size exclusion chromatography–multiangle laser light scattering (SEC–MALLS) analysis and compared them with the theoretical MW of 72 KDa for the dimeric state. All the enzymes displayed similar MW (62–65 kDa) demonstrating that they behave as dimeric species in solution (fig. 2). We recorded their CD spectra at 25 °C and after an incubation of 15 min at 80 °C. The CD spectra displayed a strong negative amplitude at 208 and 222 nm and a positive amplitude at 200 nm that reflect a typical folded protein (supplementary fig. S3, Supplementary Material online). After incubation at high temperature, there is no significant variation of amplitude at 222 nm showing that all these enzymes have remained hyperthermophilic. We concluded that mutations on residues at positions 102 and 250 did impact neither the quaternary assembly nor the apparent conformational stability. This is not surprising, because the analysis of the Wt *A. fulgidus* MalDH structure strongly suggests that the two targeted residues are not involved in intramolecular interactions.

**Fig. 3.**
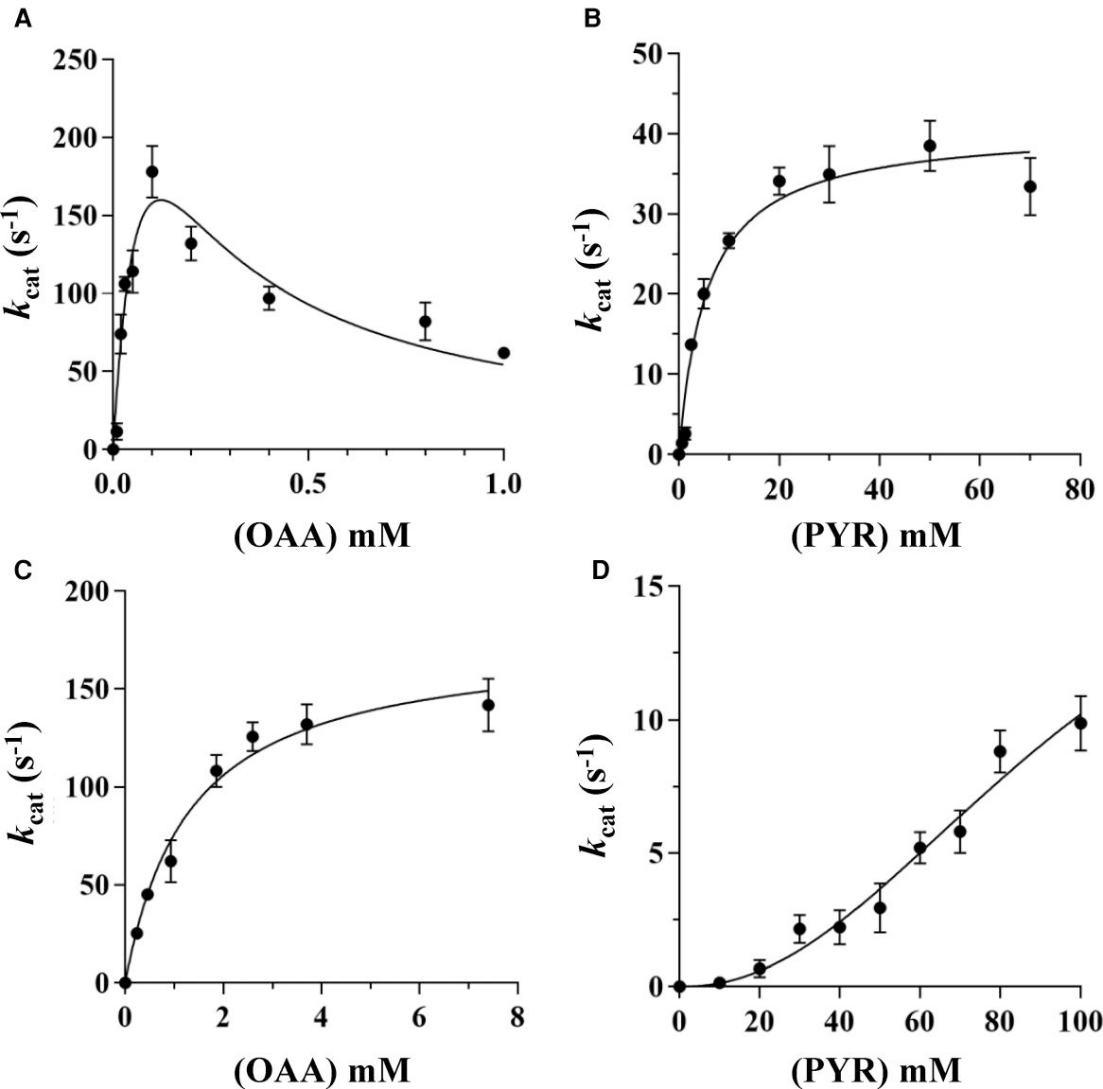
Catalytic properties of *A*. *fulgidus* MalDH and mutants at 340 K. (*A*) OAA saturation curves for the Wt enzyme carrying R102 and P250, (*B*) PYR saturation curves for the mutant 1 containing Q102 and P250, (*C*) OAA saturation curves for the mutant 2 displaying R102 and I250, and (*D*) PYR saturation curves for the mutant 3 having Q102 and I250.

In contrast, the mutations impacted functionality. Wt *A. fulgidus* MalDH displays an enzymatic activity profile that indicates inhibition by an excess of the substrate (fig. 3*A*), as it is frequently observed in many MalDHs type 3. The enzyme has a strong affinity for OAA as substrate with *K*_m_ values of 100 μM and an enzymatic efficiency in close agreement with previous reports (Langelandsvik et al. 1997). We also tested that the Wt *A. fulgidus* MalDH does not use PYR as substrate (table 1). As anticipated, the single R102Q amino acid replacement in mutant 1 changes substrate recognition from OAA to PYR (fig. 3*B*), a previously reported functional LDH mimicking transformation, which confirms that the amino acid at position 102 should be regarded as the “main specificity residue” between MalDHs and LDHs (Wilks et al. 1988; Cendrin et al. 1993; Boernke et al. 1995; Boucher et al. 2014; Steindel et al. 2016; Katava et al. 2020). However, compared with the Wt enzyme, the mutation decreases by 57-fold the affinity for the new substrate (PYR) and decreases the catalytic efficiency (*k*_cat_/*K*_m_) (fig. 3*A*and*B* and table 1). We also observed that mutant 1 is still able to recognize OAA with a lower efficiency than with PYR (table 1). This observation suggests that additional mutations are required to swap completely the specificity in MalDH toward a highly efficient LDH (Cendrin et al. 1993; Boernke et al. 1995; Yin and Kirsch 2007). It was out of the scope of this study to investigate this issue in detail.

**Table 1.** Kinetics Parameters of Wt and Mutants *A. fulgidus* MalDH.

	*K*_m_ or *S*_0.5_ (mM)	*k*_cat_ (s^−1^)	*k*_cat_/*K*_m_ (M^−1^ s^−1^)
Wt (OAA)	0.1	417	4.3 × 10^6^
Wt (PYR)	ND	ND	ND
Mutant 1 (OAA)	14	12	0.9 × 10^3^
Mutant 1 (PYR)	5.6	40	7.1 × 10^3^
Mutant 2 (OAA)	1.3	175	1.3 × 10^5^
Mutant 2 (PYR)	ND	ND	ND
Mutant 3 (OAA)	ND	ND	ND
Mutant 3 (PYR)	100	21	210

Note.—ND, not detectable.

In mutant 2, in which the initial MalDH functionality is conserved, the P250I replacement abolishes the inhibition by the excess of OAA and the activity profile exhibits a hyperbolic shape (fig. 3*C*). The affinity for OAA was significantly reduced about 31-fold with a *K*_m_ value of 1.31 mM suggesting that the P250I replacement has induced a reorganization of the binding site impacting the interaction with the substrate. We did not detect any activity in the presence of PYR with this mutant (table 1) and the Wt enzyme as it is the case with most of the MalDHs. The *k*_cat_ value of mutant 2 is lowered compared with the Wt *A. fulgidus* MalDH. Yet, the most striking change of phenotype occurred when the two mutations R102Q and P250I are combined. Indeed, the *A. fulgidus* mutant 3 exhibits a sigmoid PYR recognition profile with very high *S*_0.5_(*K*_m_) values and an important loss of catalytic efficiency (fig. 3*D* and table 1). Such a phenomenon mimics the typical behavior of many allosteric LDHs which, in the absence of their allosteric effector (FBP), exhibits sigmoid-shaped substrate saturation curves, or very low activity at physiological concentration of PYR (Arai et al. 2011). We also tested the capacity of mutant 3 to use OAA and found no activity. Yet, unlike the allosteric LDHs, there is no enzymatic activation with any of the mutants and Wt *A. fulgidus* enzyme in the presence of 0.3 and 3 mM FBP. This was expected because, at variance with the tetrameric organization, the dimeric nature of the investigated proteins does not offer appropriate binding site to FBP. In allosteric LDHs, several studies have shown that the allosteric regulation originates in the quaternary structural transition between the T-inactive state and R-active state, in which the tetramers take on extended and compact conformations, respectively (reviewed in Taguchi 2017). Our data demonstrate that, in the MalDH/LDH, just a few amino acid mutations mimicking evolutionary events may trigger homotropic activation from a nonallosteric dimeric enzyme.

### R102 and P250 Mutations Modify the Flexibility of theR171 Substrate-binding Residue of *A. fulgidus* MalDH Enzymes at Functional Temperature

In order to address the relative effect of mutations on the conformational fluctuations of *A. fulgidus* MalDH, we performed a series of molecular dynamics (MD) simulations using the Wt and mutant structural models (see Materials and Methods). We characterized the conformational landscape of Wt *A. fulgidus* MalDH, similar to the R-active state of allosteric LDHs, but as we will show, it can be altered by specific mutations toward transient conformers that mimic the T-*like* inactive state of allosteric LDHs.

**Fig. 4.**
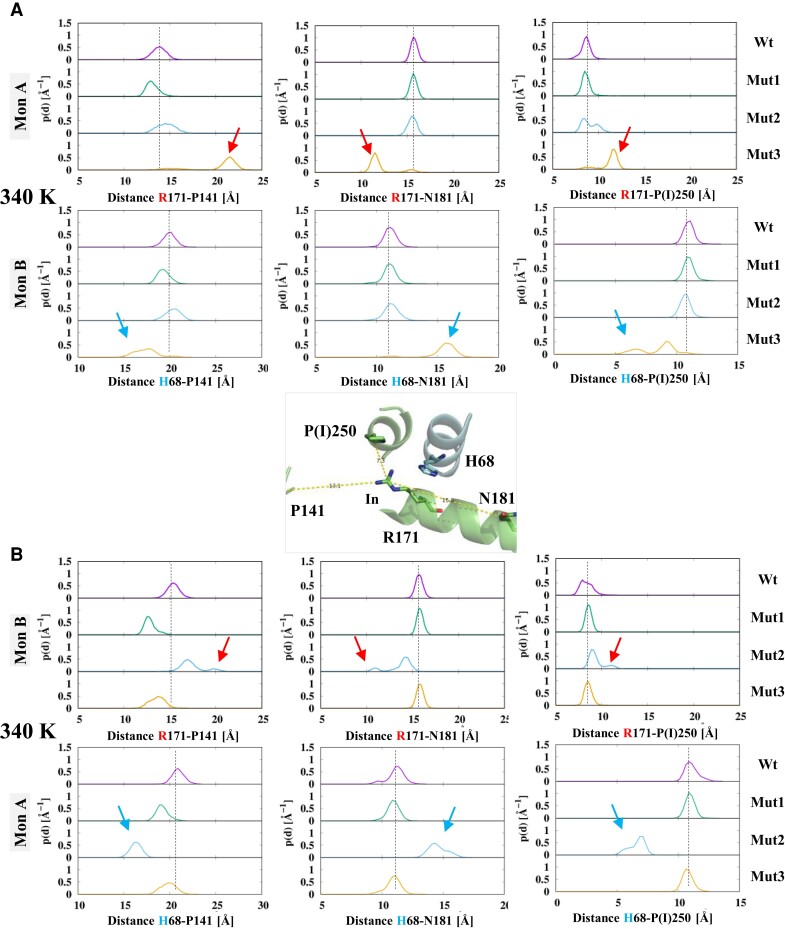
Effect of mutations on the R171 and H68 conformational substate sampling at 340 K. Probability distributions of the distances between Cγ atoms of R171 and H68 and the Cα atoms of three different residues: P141, left; N181, central; P(*I*)250, right. The inset shows the position of the considered amino acids in the crystal structure. R171 is inside (in) the catalytic site. (*A*) Fluctuations of R171in monomer A and adjacent H68in monomer B. (*B*) Fluctuations of R171in monomer B and adjacent H68 in monomer A. The dashed line refers to the distance distribution for the Wt *A. fulgidus* MalDH. The color code is purple, green, pale blue, and orange, for Wt *A. fulgidus* MalDH, mutant 1, mutant 2, and mutant 3, respectively. Arrows indicate significant distance deviations with respect to the Wt enzyme.

In order to characterize the conformational space explored by the R171 side chain, we first recorded various distances between key amino acid positions. This was done at ambient temperature (300 K) and close to the enzyme optimal functional temperature (340 K). Recall that, in a given monomer, the shorter the distance is between R171 and P141, and most of the R171 side chain is located within the catalytic site, and *vice versa* when the distance between R171 and N181 is considered (Mirror effect) (see fig. 4, close-up view). The variation of the distance between R171 and amino acid position 250 on the one hand, and the distance distribution of H68 from the adjacent monomer to the same reference amino acids at positions141, 181, and 250, on the other hand, were also monitored. At 300 K, the distance distributions are very similar among the various systems (supplementary fig. S4, Supplementary Material online), indicating that the effect of the mutations on the conformational space is not perceptible at ambient temperature.

Raising the temperature to 340 K revealed drastic changes (fig. 4). In the Wt enzyme, all the R171 and H68 distance distributions (purple lines) are quite similar indicating that each monomer behaves similarly. The distances are close to those recorded with the crystal structure, showing that 1) R171 side chains are firmly located within the catalytic site and 2) H68 is in a “closed” conformation that prevents the R171 side chain to explore the “out” conformational substates. With mutant 1(R102Q), the R171 and H68 distance distributions (green lines) are also quite similar in each monomer. Even if the R102Q mutation changes the substrate recognition property as recorded previously, this appear to be without any major consequences on the R171 and H68 local dynamics.

As a result of the P250I mutation, the distance distribution in *A. fulgidus* MalDH mutant 2 differ from those observed with the Wt enzyme. In monomer A, the impact is limited (blue lines, fig. 4*A*). The R171–P141 distance distribution is broadened and the one with I250 shows two peaks. The R171–N181 distance distribution and H68 distance distribution in monomer B were also not modified. In contrast, in monomer B, distance distributions for R171 and H68 were strongly modified (blue lines, fig. 4*B*). The R171–P141 distance distribution showed two peaks at 17 and 20 Å, whereas the “mirror” R171–N181 distribution displayed peaks at 11 and 13 Å, respectively. These measurements indicate that the R171 side chain in mutant 2 no longer protrudes into the catalytic site, as encountered in MalDHs, including the Wt *A. fulgidus* enzyme. The two peaks observed with R171–I250 shows the R171 side chain fluctuates around distinct configurations outside the catalytic site. Note that the H68 distance distributions in the adjacent monomer A are also modified. H68–P141 and H68–N181 change in opposite ways and the H68–P250 distance distribution indicates H68 has moved closer to P250. These measurements show H68 of monomer A has moved in an “open” conformational substate, which favors the R171 out substate of monomer B.

In mutant 3 that combines both R102Q and P250I mutations (orange lines, fig. 4), the effect on distance distributions is of wider amplitude. In monomer A, a major structural reorganization of R171 occurred as shown by R171–P141 values, which have increased up to 22 Å, whereas the “mirror” R171–N181 distribution has decreased by 3 Å. The values of 12 Å for the R171–I250 distance confirm a major change. As observed previously with mutant 2, the H68 distance distribution in monomer B indicates the side chain occupied an “open” position. The R171 distance distribution in monomer B indicates no major reorganization.

We concluded that the P250I mutation induces dynamically important effects on the mobility of substrate-binding residue R171. The effect is amplified, in particular at the high functional temperature when in conjunction with the mutation R102Q. In these conditions, R171 protrudes outside the catalytic site and, therefore, mimics the situation encountered in the T-inactive state of allosteric LDHs. This amino acid replacement also has a major impact on communication between monomers that alternatively can populate R- or T-like catalytic site configurations. The effect of the amino acid replacement P250I propagates to the AB monomer interface and promotes the sampling of specific conformational states of R171 and H68 side chains. Our results also provide key information about the timescale of conformational fluctuations in the two domains. In fact, we have seen that the distributions (e.g., the distances in mutant 2) are not identical between the two domains; this implies that the reshuffling among main conformations in a single domain exceeds the microsecond timescale that we probe here with our simulations.

### Impact of I250 Side-Chain Mobility on Substrate Affinity

With the P250I mutation, a nonpolar side chain protrudes in the catalytic site. With mutant 3, which carries the additional R102Q mutation, MD simulation at 340 K reveals that the I250 side chain visits three configurations (Cs1, Cs2, and Cs3) (supplementary fig. S5*A*, Supplementary Material online). As expected, the superposition between the Wt enzyme and the most representative conformers for I250 side chain indicates its location within the catalytic site close to the reactive extremity of NADH (supplementary fig. S5*B*, Supplementary Material online). The comparison with representative structures of the allosteric *T. thermophilus* LDH in the T- and R-states indicates that Cs3 mimics the T-like state (supplementary fig. S5*C*, Supplementary Material online, right panel), whereas Cs2 corresponds to the R-like state (supplementary fig. S5*D*, Supplementary Material online, central panel). Because of a putative steric clash between the NADH reactive extremity (C4 ring carbon) and I250 Cs1, that would hinder the correct geometry requested for an efficient hydride ion transfer, we considered this substate as unfavorable (supplementary fig. S5*D*, Supplementary Material online, left panel). We observed that I250 is connected to T246 through van der walls contact with the main chain of amino acid at position 251. Amino acid at position 246 also participates interaction with the substrate (Brochier-Armanet and Madern 2021). T246 conformational substates in *A. fulgidus* MalDH mutant 3 differ from those observed in the R-active state of an allosteric LDH (supplementary fig. S5, Supplementary Material online). Consequently, in addition to the capacity of R171 to explore a T-like state, none of the favorable conformational substates combination between I250 and T246 are achieved to ensure strong PYR affinity and high catalytic efficiency in mutant 3.

### Mutation of R102 and P250 modify the conformational landscapes of *A. fulgidus* MalDH at functional temperature

**Fig. 5.**
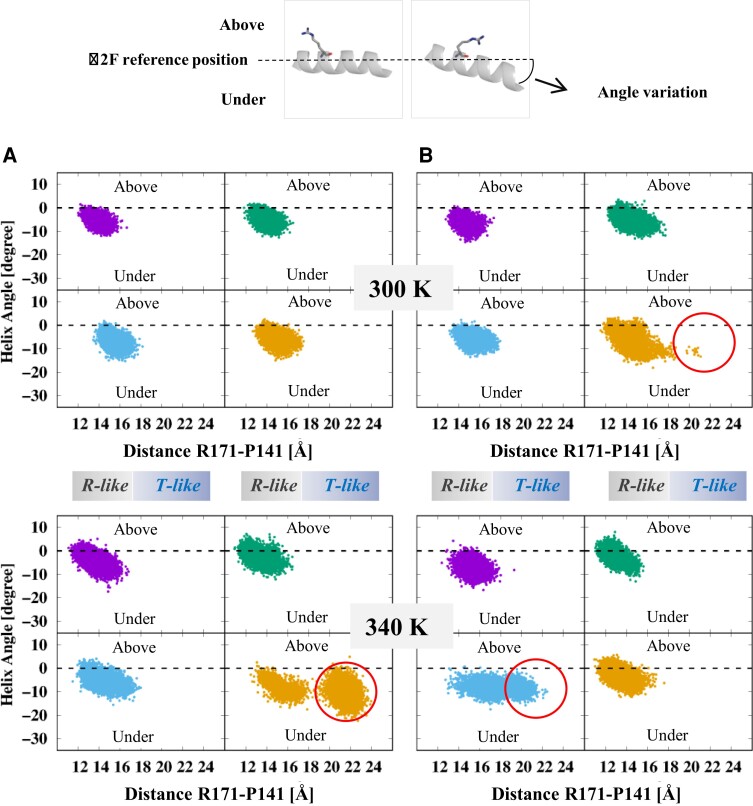
Conformational landscape variation of the α2*F* helix in *A. fulgidus* MalDH and mutants. The color code is purple, green, pale blue, and orange, for Wt *A. fulgidus* MalDH, mutant 1, mutant 2, and mutant 3, respectively. (*A*) Monomer A at 300 K (top) and 340 K (bottom). (*B*) Monomer B, same conditions. The top close-up view corresponds to the various α2*F* helix positions as observed in the *T. thermophilus* LDH crystal structures representative of the R- and T-states, left and right images, respectively.

We now focus our attention on one helix, which displays an important conformational reorganization in allosteric LDHs (Iwata et al. 1994; Coquelle et al. 2007). In the LDH structure representative of the R-active state (PDB accession number 2V7P), the α2F helix is in a rather “flat” position, as illustrated in fig. 5. The substrate-binding residue R171 is in a favorable position to interact with PYR within the catalytic site. In the LDH structure representative of the T-inactive state (PDB accession number 2V6M), the α2F helix moves “under” with respect to its position in 2V7P and the side chain of R171 is projected outside the catalytic site (Iwata et al. 1994; Coquelle et al. 2007).

We analyzed whether or not the WT enzyme and mutants were adopting 2V6M-like α2F helical angles during the simulations, and found it was not the case with the exception of mutant 3 (supplementary fig. S6, Supplementary Material online). Then, we drew a 2D scatter plot to analyze the change in the conformational landscape due to the mutations (fig. 5). It is interesting to note that at 300 K, all the enzymes behave in the same way in each monomer. However, in mutant 3, monomer B explores different conformations (red circles). At 340 K, the temperature increase allows a more drastic change in mutants 2 and 3 that carry the P250I mutation. In mutant 2, the scatter plot profile for monomer A is not impacted. In contrast, with monomer B, the plot is ellipsoidal showing the number of conformers with both a R171-out and a α2F “under” configuration has strongly increased (red circles). With mutant 3, the monomer A profile shows two separate clouds indicating that conformers with the R171-out and a α2F under configuration are strongly favored (red circles). As with mutant 2, the adjacent monomer profile is not impacted.

**Fig. 6.**
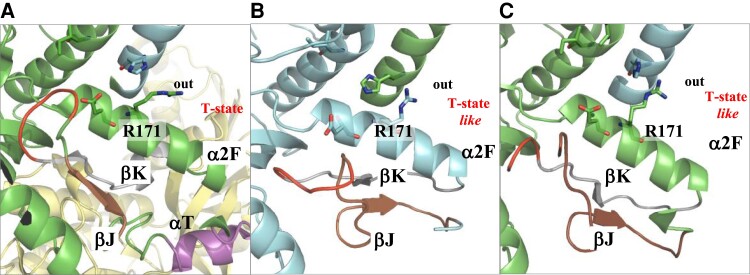
Structural comparison between mutants 2 and 3 of *A. fulgidus* MalDH, with *T. thermophilus* LDH T- state (PDB ID 2V6*M*). (*A*) Close-up view of *T. thermophilus* LDH). Monomers A, B, and D are in green, cyan, and yellow, respectively. Monomer C is not shown. R171 is outside the catalytic site (out). Several secondary structure elements are identified. (*B*) Conformers of *A. fulgidus* MalDH mutant 2, from MD simulation at 340 K, “189,500 ps.” (*C*) Conformers of *A. fulgidus* MalDH mutant 3, from MD simulation at 340 K, “750,000 ps.”

We selected two conformers of mutants 2 and 3 along the MD simulations, which displayed strong parameter deviations (red circle in fig. 5) with respect to those of the Wt enzyme, and superimposed their structures with the representative crystal structure of an allosteric LDH in the T-inactive state (fig. 6). The apo state LDH from *T. thermophilus* was used (Coquelle et al. 2007).

The structural comparison shows that some conformers of mutants 2 and 3 at 340 K display a local reorganization of the α2F helix and R171 that resembles the typical structure of an allosteric LDH in the T-state. These data demonstrate that the P250I mutation is of major importance in changing the dynamical properties of *A. fulgidus* MalDH. Not only does it allow R171 to explore a noncompetent state for substrate binding, but it also promotes correlated motions between residues at the A–B interface. Note that the capacity of I250 to promote an allosteric-like effect as in LDH is strongly amplified when the mutation R102Q, allowing to change functionality, is added.

### Effect of Mutations on the Flexible Active Site Loop

**Fig. 7.**
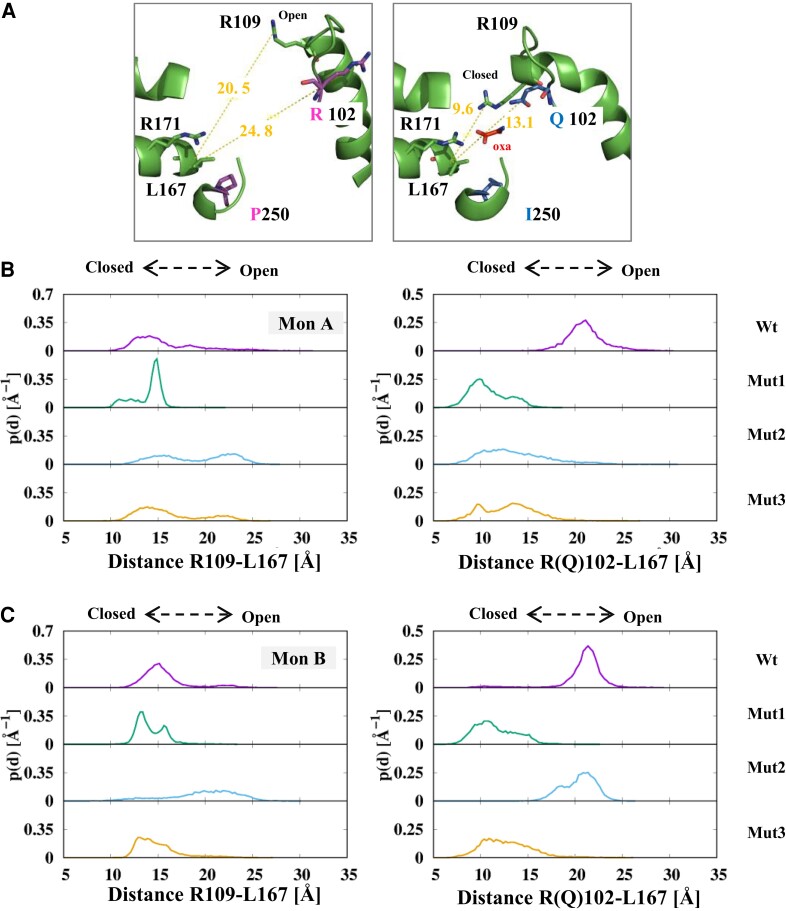
Mobile loop fluctuations in Wt *A. fulgidus* MalDH and mutants at 340 K. The figure shows the probability distributions of the distances between CG atoms of R(*Q*)102 and R109 and the Cα atoms of L167. (*A*) The left and right small insets show the position of the various amino acids considered in *A. fulgidus* MalDH and *T. thermophilus* LDH crystal structures, respectively. (*B*) Fluctuations in monomer A. (*C*) Fluctuations in monomer B. The color code is purple, green, pale blue, and orange, for Wt *A. fulgidus* MalDH, mutant 1, mutant 2, and mutant 3, respectively.

In numerous crystal structures of LDHs and MalDHs, the active site loop that carries the substrate discriminating amino acid at position 102 has been described in two different conformations (Iwata et al. 1994; Auerbach et al. 1998; Winter et al. 2003; Coquelle et al. 2007; Arai et al. 2010; González et al. 2018). These correspond to either open or closed conformations, as observed in the Apo or Holo structures, respectively. The open position is shown in figure 7*A*. It corresponds to the crystal structure of Wt *A. fulgidus* MalDH. In this case, the R102 and R109 side chains are far away from the catalytic crevasse.

Their distances with respect to the α2F helix extremity, monitored using the conserved position L167, are larger than 20 Å. The typical closed position for the loop is observable in the crystal structure of *T. thermophilus* LDH in the Holo state (fig. 7*A*). In this case, the side chains of R109 and Q102 are located closer to the α2F extremity, with values 9.6 and 13.1 Å, respectively. Note that in numerous structures, the loop is not modeled because of a too high flexibility.

We monitored these distances in the simulations of the Wt *A. fulgidus* MalDH and mutant systems (fig. 7*A*and*B*). At 340 K, for each monomer, in the Wt enzyme, the distance L167–R102 shows that the R102 side chain remains far away from the catalytic site, whereas the L167–R109 distance (around 15 Å) indicates a situation slightly different from the crystallographic structure suggesting a local distortion that favors the approach of R109 toward the catalytic site. In mutant 1, for both monomers, the distance distribution for L167–R109 is globally unchanged with a distribution in a range of 13–15 Å. In contrast, the L167–Q102 distribution is shifted toward the lowest values than in the Wt enzyme, a situation that mimics the situation observed in the LDH crystal structure when the substrate analog is bound (Holo state). In mutant 2, the L167–R109 distribution shows two peaks in monomer A, indicating that the side chain fluctuates between open and closed conformations. In monomer B, the R109 is in a rather open position. In monomer A, the L167–R102 distance distribution agrees with a closed conformation for R102. In contrast, the side chain of R102 occupies the open position in monomer B. In mutant 3, the distance distribution that accounts for R109 and Q102 positions suggests that their side chains are in a closed-like conformation.

Our data suggest that in the Wt and mutants of *A. fulgidus* MalDH, fluctuations of the mobile loop in the Apo state are strongly constrained by electrostatic interactions between R(Q)102, R109, and R171. When R102 is present, a dominant repulsive regime between three positive charges maintains the loop in an open-like state. With Q102, the charge repulsion effect is low and the loop can more easily explore the closed-like state. The data also show that the loop movements are better described by a twist rather than by a rigid translation.

## Discussion

Strongly inspired by the pioneering concept linking the capacity of proteins to evolve and conformational dynamics proposed by Tokuriki and Tawfik (2009), we addressed the question concerning the origin of allostery in LDH.

Allosteric regulation in LDHs fits the MWC model (Monod et al. 1965), in which an allosteric enzyme fluctuates (even in the absence of the effector) between at least two conformational states—R (relaxed, with low affinity for the substrate) or T (tense, with high substrate affinity) (Taguchi 2017 and reference therein). Apo and Holo crystal structures of LDHs representative of the T- and R-states show that the position of the side chain of the strictly conserved R171, a relevant proxy for the T- or R-states (Iorio et al 2021), is always found either outside or inside the catalytic site of all monomers.

**Fig. 8.**
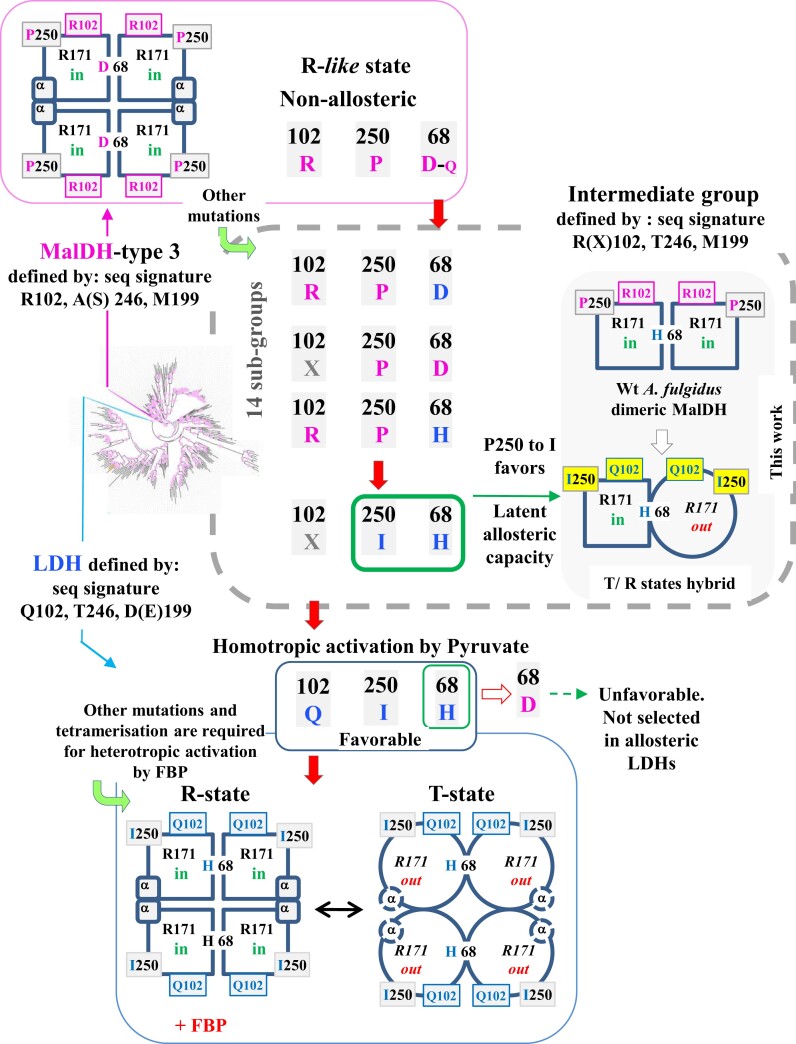
Scheme of functional and dynamical evolution associated with the emergence of canonical LDHs from MalDH type 3. The three mains groups of enzymes (MalDH type 3, canonical LDH, and intermediate enzymes) are defined according to phylogenetic analyses (Brochier-Armanet and Madern 2021). The evolution of amino acid at key positions (i.e., 68, 102, and 250) is indicated. The active (R-) an inactive (T-) state for a monomer in a given enzyme is represented by a square and a circle, respectively. An amino acid colored in pink or in blue indicates a MalDH or an LDH characteristic, respectively. *In* and *out*, refers to the R171 side-chain position within or outside the catalytic site, respectively. The red arrow shows the trajectory of key amino acid replacements requested to facilitate the evolution of nonallosteric MalDH toward LDH, in which allosteric regulation can emerge. The inset in gray summarized the effect of the P250*I* mutation probed in this work. The consequence of H68*D* mutation in an allosteric LDH is indicated.

First, by combining a phylogenetic and structural analysis, we suggested that amino acid replacements of two amino acid positions (68 and 250) located within the catalytic pocket would have had a strong influence on the transition between nonallosteric MalDHs type 3 and canonical allosteric LDHs. Our phylogenetic analysis, based on Brochier-Armanet and Madern (2021), showed the predominant occurrence of H68 and D68 in the intermediate group of MalDHs, suggesting different abilities regarding allostery (fig. 8), with a preexisting capacity in enzymes harboring H68 prior to the fixation of this amino acid in LDHs. LDH crystal structures showed that the conformational fluctuations of R171 rely on the presence of H68 that sustains the flexibility at the monomer A–monomer B interface, and allows the R171 side chain to swing out of the catalytic site. In canonical LDH, the replacement of H68 by D68 contributes to a salt-bridge formation with R171, which abolishes the sigmoid activity profile of the allosteric bacterial LDH mutant from *Lactobacillus casei* (Arai et al. 2011). The crystal structure of nonallosteric LDHs from *Lactobacillus pentosus* (PDB code: 1EZ4) indicates a D at position 68 that also maintain R171 in the R-active position (Uchikoba et al. 2002). In nonallosteric MalDH type 3, representative crystal structures (PDB code: 4CL3, 4BGV) show that when a negatively charged residue (E68) establishes a salt-bridge with R171, its side chain protrudes within the catalytic site. MDS simulation using 4CL3 did not reveal any changes from the R-active toward the T-inactive state at high functional temperature (Kalimeri et al. 2014). These observations demonstrate that the presence of a histidine at position 68 strongly contributes to establish the allosteric phenotype.

Second, we analyzed the consequence of the P250I substitution in an enzyme from the intermediate group that carries H68, with *A. fulgidus* MalDH as a model. Here, the P250I mutation has allowed the I250 side chain to sample several conformations within the catalytic site, which mimic those existing in the T- and R-states of an allosteric LDH. Several studies have shown that the proline could be seen as a small switch that modulates the allosteric behavior (Collins et al. 1995; Rodicio et al. 2000; Weyand et al. 2002; Vogel et al. 2006; Wei et al. 2014). Because the proline ring restricts the protein backbone flexibility, its replacement by another amino acid is not neutral. This mutation should be considered as a dynamics-enhancing replacement that rendered enzymes from the intermediate group prone to evolve toward allostery prior to its fixation in canonical LDH.

Third, we observed that while the *A. fulgidus* MalDH mutant 2 differs from the Wt enzyme only by one amino acid (P250I), it exhibited a lower affinity for OAA. A strong decrease in affinity for PYR was also detected in *A. fulgidus* MalDH mutant 3. We analyzed by MDS that introducing I250 side chain in the catalytic site has an unexpected unfavorable consequences of the T246 conformers sampling, a residue also involved in substrate discrimination that should favor PYR recognition (Brochier-Armanet and Madern 2021, reference therein). It confirms the hypothesis done by Brochier and Madern (2021), which stipulates that the appearance of I250 together with the A/S to T replacement at position 246 in enzymes of the intermediate group, contributed to the extinction of OAA recognition during the MalDH to LDH functional transition. We suggest that the *A. fulgidus* mutant 3 (P250I and R102Q) low catalytic efficiency and low substrate affinity are caused by an increased number of unproductive conformers of the active site related to I250 fluctuations. Very recently, it has been shown that unfavorable conformations of amino acid side chains can drive major structural reorganization in a JNK interacting protein (Pérez et al. 2022).

Fourth, we notice that these two substitutions occurred prior to the critical replacement of R at position 102 by Q in the stem of canonical LDH clade, considered the main discriminating residue for functionality between MalDH and LDH (Wilks et al. 1988; Cendrin et al. 1993; Boernke et al. 1995; Boucher et al. 2014; Steindel et al. 2016; Katava et al. 2020; Brochier-Armanet and Madern 2021). Therefore, we assume that owing to its pleiotropic effect, the amino acid replacement P250I was one of the strongest drivers of phenotypic evolution within the MalDH/LDH superfamily. Notice that the “allosterized” capacity of the *A. fulgidus* induced by P250I was enzymatically detectable only in the presence of the extra R102Q mutation that acts at the level of substrate recognition. In addition, by using MDS, we revealed that electrostatic property changes, induced by the R102Q mutation, promoted a structural distortion of the mobile binding site loop that modulates the open/closed propensity in the Apo state. We propose that this mutation not only serves functional evolution, but in combination with I250, enlarges the conformational landscape of the protein. The observation is in agreement with a work showing that allosteric propensity is coupled with loop dynamics (Galdadas et al. 2021). Yet, the combination on a MalDH scaffold of H68, Q102, T246, and I250 was not sufficient to design a homeotropically activated LDH with high catalytic efficiency. This is likely due to the phenomenon of epistasis, that is, the context-dependence of mutation in the relationship between sequence changes and phenotypic variation (Poelwijk et al. 2016), meaning that additional changes are required to fully achieve the MalDH/LDH functional transition. Here, the shorter sequence of *A. fulgidus* MalDH, compared with most of its homologs, has reorganized the structure (see below) so that the complete putative effect of allosteric LDH-like mutations was not fully transferable. Studies have shown that the accumulation of amino acid substitutions allows the recruitment and optimization of a preexisting low-level property in a protein scaffold, creating proteins with high latent evolutionary potential (i.e., marked ability to evolve new properties) (see Khersonsky and Tawfik 2010 and references therein). The potentiality to evolve these enzymes with latent properties can be controlled either by a relaxed or by a stringent negative tradeoff between the initial and final function (Khersonsky and Tawfik 2010). This then allows the promiscuous intermediate to change their properties with very few amino acid substitutions (Thornton et al. 2003; Wouters et al. 2003; Zou et al. 2015; Otten et al. 2018). The various steps that relate the MalDH/LDH evolution fit well these fundamental principles.

Fifth, our data based on the MalDH/LDH superfamily add precious information for understanding the evolution of allostery. By exploiting the resolution of MDS, we found that, by introducing two LDH-like mutations into a dimeric archaeal MalDH from the intermediate group, the resulting protein homotropicaly activated by PYR starts visiting, even in the absence of any ligands, new configurational states relevant for allosteric regulation (see the gray inset in fig. 8). This few mutations could be considered as permissive for the selection of a new phenotype as previously observed with other allosteric enzymes (Kuo et al. 1989; Stebbins and Kantrowitz 1992; Farsi et al. 2012). Some studies suggested that the allosteric property of modern proteins arose in ancestral proteins with a latent capacity for allosteric modulation (Reynolds et al. 2011; Coyle et al. 2013; Hadzipasic et al. 2020), while others proposed that allosteric regulation emerged from nonallosteric extinct ancestral enzymes (Mas-Droux et al. 2006; Raman et al. 2016; Schupfner et al. 2020). Two studies based on ancestral sequence reconstruction have analyzed the selection of allostery in contemporary PYR kinases and steroid receptors. This is due to mutations at some key positions that exist within interaction networks and which, may rewire the network, subsequently altering the transmission of the signal (Johnsen et al. 2019; Okafor et al. 2020). Different studies provided convincing explanations to explain why few mutations are responsible for changes in allosteric capacity. This is because, in enzymes, the dynamical process is hierarchical and covers different time scales that influence functionality and allostery (Frauenfelder et al. 2001; Wrab et al. 2011; Choy et al. 2017; Fleishman and Horovitz 2021; Wolf et al. 2021; Madhu et al. 2022; Makurat et al 2022). Because of this hierarchy, very short time scale dynamics, due to simple side-chain fluctuations of a single residue can influence reorganization between the T- and R-states (Zhou et al. 2003; Fujiwara et al. 2017; Iyer et al. 2021). How the enzymatic activation (heterotropic activation) of LDHs by the allosteric effector (FBP) evolved (concomitantly or successively) requires additional investigations.

Finally, we discuss the allosteric capacity in MalDH and LDH superfamily in light of current concepts of allostery. The initial descriptive phenomenological models of allostery are the MWC and KNF models (Monod et al. 1965; Koshland et al. 1966). In the former, all monomers of the oligomeric enzyme are in the same state, being either T- or R-; whereas in the latter that may exist as a mixture of both states. Several structural studies based on the Wt states of allosteric LDHs have suggested that they obey the MWC model in which all monomers are in the same state (Iwata et al. 1994; Auerbach et al. 1998; Winter et al. 2003; Coquelle et al. 2007; Arai et al. 2010). However, by introducing mutations in the hinge regions of allosteric *T. thermophilus* LDH, it has been possible to observe a strong structural heterogeneity between monomers in crystal structures caused by a change in enzyme flexibility (Colletier et al. 2012). The “allosterized” *A. fulgidus* mutant 2 behavior agrees with the phenomenological KNF descriptive model of allostery, in which ligand binding induces sequential conformational changes within the oligomeric enzyme that may exist as a mixture of T- and R-states (Fersht 1999). The differences we observed are not antagonistic. Indeed, in the recent and integrative ensemble model of allostery, the allosteric character of an enzyme is determined by the redistribution of the occupancy probabilities of different conformational states (Wrabl et al. 2011; Motlagh et al. 2014). We propose that the different apparent behaviors are due to different oligomeric states of LDH, which are always tetrameric, while the “allosterized” *A. fulgidus* mutants are dimeric. Indeed, in the dimeric *A. fulgidus* MalDH, there is a 6 amino acids deletion that prevents the formation of an equivalent secondary structural element (see αT, in fig. 6) as in tetrameric MalDHs or LDHs. In allosteric LDHs, this helix contributes to (1) the allosteric core and (2) the FBP-binding site formation, and also helps to establish the interface, between the dimer of dimers that glues the tetrameric scaffold (Auerbach et al. 1998; Coquelle et al. 2007; Iorio et al. 2021). In allosteric LDHs, mutations that target this helix have a strong influence on the *R*–*T* equilibrium (Colletier et al. 2012). We propose that the presence or absence of this structural element drastically controls the propagation of the changes induced by mutations favoring allosteric regulation in LDHs. In a recent study, Brochier-Armanet and Madern (2021) proposed that the dimeric state of the enzyme from the intermediate group is the minimal functional unit in which the homotropic activation has evolved; our present study demonstrated the case. Heterotropic activation, on the other hand, requires the tetrameric scaffold.

## Materials and Methods

### Phylogenetic Analyses

The multiple alignment and the maximum likelihood phylogeny of the LDH/MalDH type 3 part of the LDH/MalDH superfamily have been obtained from Brochier-Armanet and Madern (2021). The mapping amino acids residues at positions 68, 102, 246, and 250 were on the tree was performed using iToL v.6.4 (Letunic and Bork 2021).

### Cloning of *A. fulgidus* MalDH

A gene encoding the malate dehydrogenase of *A. fulgidus* (NCBI Reference Sequence: WP_010878358.1) with codons optimized for expression in *Escherichia coli* was synthesized and subcloned into pET-20a by Gencust. The various mutants used in this study were also constructed by the supplier. The NdeI and BamHI cloning sites were used for cloning.

### Protein Expression and Purification

1. *Escherichia coli* BL21 DE3pLyS competent cells transformed with pET-20 expression vector encoding *A. fulgidus* MalDH gene, and mutants were selected by growth on LB agar plates containing 100 µg ml^−1^ ampicillin. A single colony was grown overnight at 37 °C in 50 ml^−1^ LB medium at the same concentration of antibiotic. Twenty millilitres of these cultures were then used for inoculation of a 2 l LB medium containing 100 µg ml^−1^ ampicillin. The cells were cultivated at 37 °C until an OD_600_ of 0.6 was reached. Isopropyl B-d-1-thiogalactopyranoside (IPTG) was added at a final concentration of 0.5 mM to induce expression and the culture was incubated for 4 h at 37 °C. Bacterial cells were harvested by centrifugation at 6,000 × g for 20 min at 4 °C. The pellet was suspended in 40 ml of 50 mM Tris–HCl pH 7.4 containing 50 mM NaCl (Buffer A). Prior to cell disruption, 5 µg ml^−1^ of DNAse and MgCl_2_ to the final concentration of 10 mM was added to the cell suspension. The preparation was cooled at 4 °C and lysed by sonication (Branson sonicator). Six cycles of continuous sonication at 50% amplitude were applied during 30 s. Between each pulse, the solution was kept on ice for 1 min. The extract was then centrifuged at 13,000 × g for 30 min at 4 °C. The supernatant was further incubated at 70 °C for 30 min and the thermally unfolded proteins were removed by centrifugation. The extract was loaded on a Q sepharose column (2 × 10 cm) equilibrated with Buffer A. *Archaeoglobus fulgidus* MalDH and mutants were eluted with a linear gradient from 0 to 0.8 M NaCl in Buffer A. The active fractions containing the enzyme were pooled, concentrated, and loaded on a Superpose 12 gel-filtration column (GE Healthcare) and eluted with Buffer A. The purity of enzymes was checked by SDS gel electrophoresis. The enzymes were concentrated at 20 mg ml^−1^ in Buffer A and stored at 4 °C.

### Enzymatic Assay and Protein Determination

The reduction of oxaloacetate to malate of *A. fulgidus* MalDH and mutants was measured in 500 µl of 50 mM K_2_HPO_4_/KH_2_PO_4_ pH 7.0 and supplemented with 50 mM NaCl. The reduction of PYR to lactate was determined in 500 µl of 2-(*N*-morpholino) ethane sulfonic acid (MES) pH 6.0 supplemented with 50 mM NaCl. The reaction was monitored spectrophotometrically at 340 nm by following the oxidation of NADH (0.5 mM) on a Jasco 540 at 70 °C. To record the enzymatic profile of both enzymes, various substrate concentrations were tested. The data were analyzed using Michaelis–Menten or allosteric sigmoidal equations in GraphPad Prism version 7.03. The protein concentration was estimated from the absorbance at 280 nm using a Nanodrop 2000 (Thermo Scientific), with molecular weight and extinction coefficient calculated using the server https://web.expasy.org/protparam.

### Circular Dichroism

Far-UV CD measurements were carried out on a JASCO J-810 thermostated spectropolarimeter. Far-UV spectra were recorded in 0.10 cm path length quartz cells. The spectra shown in this work represent the average of three accumulated consecutive scans. The protein concentration was 0.3 mg ml^−1^.

### Determination of Native Molecular Masses of the Purified Enzymes

SEC was carried out with a flow rate of 0.5 ml min^−1^ on an ENrich™ SEC650 10 × 300 gel-filtration column (BioRad). Calibration of the column was performed with the gel filtration standard from BioRad. Direct comparison of the elution profiles was achieved using the Compare mode of the BioLogic FPLC operating system (BioRad).

### Size Exclusion Chromatography–Multiangle Laser Light Scattering

SEC combined with online detection by MALLS and refractometry (RI) was used to measure the absolute molecular mass of proteins in solution. The SEC run was performed using an Enrich 650 column (BioRad) equilibrated with a buffer composed of 50 mM Tris–HCl pH 7.2 and 50 mM NaCl. Separation was performed at room temperature and 50 μl of protein sample, concentrated at ∼1 mg ml^−1^, was injected with a constant flow rate of 0.5 ml^−1^ min^−1^. Online MALLS detection was performed with a DAWN-HELEOS II detector (Wyatt Technology Corp.) using a laser emitting at 690 nm. Protein concentration was determined by measuring the differential refractive index online using an Optilab T-rEX detector (Wyatt Technology Corp.) with a refractive index increment *dn/dc* of 0.185 ml^−1^ g^−1^. Weight-averaged molecular weight (*M*_w_) determination was done with the ASTRA6 software (Wyatt Technologies) and the curve was represented with GraphPad Prism.

### Molecular Dynamics

We used the approach that we developed in a previous study aimed to analyse the thermal-dependent allosteric behavior of two LDHs (Iorio et al. 2021). All-atom MD simulations of the Wt *A. fulgidus* MalDH and three mutants were performed. The simulations were started from the crystallographic structure (2X0I) and homology-based models. Structural models of *A. fulgidus* mutants were generated using the Swiss model server (https://swissmodel.expasy.org/) (Waterhouse et al. 2018). Careful inspection of the crystallographic structure and the homology models did not reveal significant differences. All the simulations were done using the APO states and were carried out in the NPT ensemble at ambient pressure, 1 atm, using the GROMACS 2018 software (Vander Spoel et al. 2005; Abraham et al. 2015), the Charmm36 force field for proteins (Huang et al. 2017), and the TIP3P three points water model. Ions were added to the simulation boxes to neutralize the systems. After the equilibration phase, the systems were allowed to evolve at two temperatures, 300 and 340 K, for 1 μs with a time step of 2 fs.

Two-dimensional scatter plots were obtained in the following way. We first aligned α2F of the Wt enzyme (PDB accession number 2XOI) with the *X*-axis of the simulation box; this allows us to create a reference state. The orientation of the helix was given by the vector joining Cα atoms of residues 167 and 181 (Normalized numbering), taken as the *extrema* of the helix. Then, by using the Cα atoms of all residues in a monomer, we aligned each monomer in the MD trajectory with the crystallographic structure of the reference state using a fitting procedure. The angle between the helix in the MD trajectories and the reference state (from the *X*-axis aligned reference structure) is defined as that formed by the projection of the helix vector in the MD simulations on the *X*–*Z* plane with the reference vector of the crystallographic structure. The computed values of the helix angle and the R171–P141 distance were combined to draw a scatter plot that is useful to recognize the conformational changes representative of the R- or T-like states.

## Supplementary Material

msac186_Supplementary_Data

## Data Availability

The data are available in the article and its online Supplementary material. The MDS trajectories are accessible at https://zenodo.org/record/6380887#.YjxRATXjKUk.
