# Supplementary material for: Protein Conformational Space at the Edge of Allostery: Turning a Nonallosteric Malate Dehydrogenase into an “Allosterized” Enzyme Using Evolution-Guided Punctual Mutations
Source: Mol Biol Evol. 2022 Sep 3;39(9):msac186. doi: 10.1093/molbev/msac186 (PMC9486893; doi:10.1093/molbev/msac186)
Supplement: msac186_Supplementary_Data [file msac186_supplementary_data.pdf]

Supplementary Material.

Protein Conformational Space at the Edge of Allostery: Turning a Non-allosteric Malate Dehydrogenase into an "Allosterized" Enzyme using Evolution Guided Punctual Mutations

Antonio Iorio[1], Céline Brochier-Armanet[2], Caroline Mas[3], Fabio Sterpone[1]*and Dominique Madern[3]*

1- CNRS, Université de Paris, UPR 9080, Laboratoire de Biochimie Théorique, Paris, France; Institut de Biologie Physico-Chimique-Fondation Edmond de Rothschild, PSL Research University, Paris, France.

2- Univ Lyon, Université Lyon 1, CNRS, UMR5558, Laboratoire de Biométrie et Biologie Évolutive, 43 bd du 11 novembre 1918, F-69622, Villeurbanne, France.

3-Univ. Grenoble Alpes, CEA, CNRS, IBS, 38000 Grenoble, France

* Corresponding authors:

Dr Dominique Madern, Dr Fabio Sterpone,

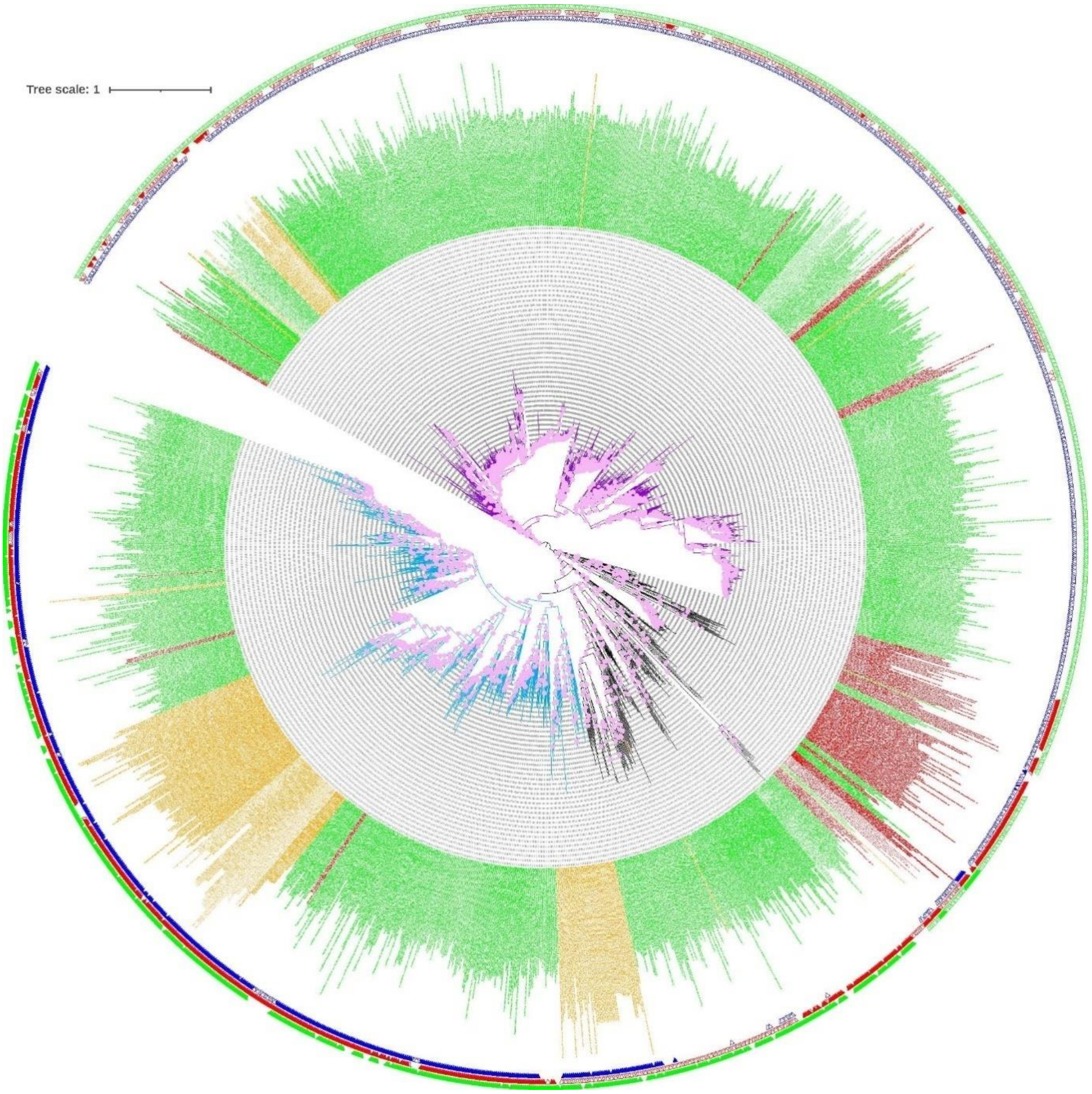

Supplementary Figure S1. Unrooted maximum likelihood tree of the LDHs, MalDH type 3, and intermediate MalDHs.

The tree was inferred with the 1,635 sequences present in 2,272 representative proteomes as described elsewhere (Brochier-Armanet and Madern 2021). Sequences from Archaea are represented in red, Bacteria in green and Eukarya in orange. The scale bar represents the average number of substitutions per site. Circles at branch correspond to ultrafast bootstrap values. For clarity, values lower than 90% were omitted.

LDH sequences (blue branches) harbor mainly the Q102 (filled blue triangles), I250 (filled red triangles), and H68 (filled green triangles) signature, while MalDH type 3 sequences (pink branches) contain mainly R102 (empty blue triangles), P250 (empty red triangles), and D/Q68 (empty green triangles). Sequences of the MalDH intermediate group display mixed combinations of the LDH and MalDH type signatures.

```
                                   sssss   hhhhhhhhhhhhhhh   sssss  hhh  hhhhhhhhhhhhh
1-MalDH              -----MARSKIALIGA-GQIGGTLAHLAGLKEL-GDVVLFDIVDG--VPQGKALDIAESA    51
2-MalDH              ------MRKKISIIGA-GFVGSTTAHWLAAKEL-GDIVLLDIVEG--VPQGKALDLYEAS    50
3-MalDH              -----MARNKIALIGS-GMIGGTLAHLAGLKEL-GDVVLFDIAEG--TPQGKGLDIAESS    51
4--intermediate      --------MKVTIIGASGRVGSATALLLAKEPFMKDLVLIGREHSINKLEGLREDIYDAL    52
5--intermediate      -------MSKVAVIGATGRVGSTAAARLALLDCVNEVTLIARPKSVDKLRGLRRDILDSL    53
6--intermediate      ---MARIPYKVAVIGT-GRVGATFAYTMAVVPGIARMTLVDVVPG--LAKGVMEDIKHAA    54
7--intermediate      --------MKLGFVGA-GRVGSTSAFTCLLNLDVDEIALVDIAED--LAVGEAMDLAHAA    49
8--intermediate      ----MNNRRKIVVIGA-SNVGSAVANKIADFQLATEVVLIDLNED--KAWGEAKDSSHAT    53
9--intermediate      ----MIKKRKVVIVGA-GNVGASTAFCMINQGICDEIVLIDMNTE--RAKGETLDMIHSI    53
10-intermediate      -------ARKIGIIGL-GNVGAAVAHGLIAQGVADDYVFIDANEA--KVKADQIDFQDAM    50
11-LDH               --------MKVGIVGS-GMVGSATAYALALLGVAREVVLVDLDRK--LAQAHAEDILHAT    49
12-LDH               MAETTVKPTKLAVIGA-GAVGSTLAFAAAQRGIAREIVLEDIAKE--RVEAEVLDMQHGS    57
13-LDH               --MKNNGGARVVVIGA-GFVGASYVFALMNQGIADEIVLIDANES--KAIGDAMDFNHGK    55
                                                                            68

                     hhhh   sssssh  hhhhhh   ssss               hhhhhhhhhhhhhhhhhh
1-MalDH              PVDGFDAKYSGAS--DYSAIAGADVVIVTAGVPRKPGM----SRDDLIGINLKVMEAVGA   105
2-MalDH              PIEGFDVRVTGTN--NYADTANSDVIVVTSGAPRKPGM----SREDLIKVNADITRACIS   104
3-MalDH              PVDGFDAKFTGAN--DYAAIEGADVVIVTAGVPRKPGM----SRDDLLGINLKVMEQVGA   105
4--intermediate      AGTRSDANIYVESDENLRRIIDESDVVIITSGVPRKEGM----SRMDLAKTNAKIVGKYAK   108
5--intermediate      AAAQKDAEITIGCERDD--YVDADVIVMTAGIPRKPGQ----TRLDLTKDNAAIIKKYLE   107
6--intermediate      AVFRRSITVEAFE--DVSKVENADAIVITAGKPRKADM----SRRDLANVNAQIIRDIGD   108
7--intermediate      AGIDKYPKIVGGA--DYSLLKGSEIIVVTAGLARKPGM----TRLDLAHKNAGIIKDIAK   103
8--intermediate      SCIYSTNIKFHLG--DYEDCKDANIIVITAGPSIRPGE--TPDRLKLAGTNAKIMSSVMG   109
9--intermediate      A-FMNRNMQIKLG--DYTDCKDADVLVITASAPMGKEN----DRLVALKSSSKIVTSIVS   106
10-intermediate      ANLE-AHGNIVIN--DWAALADADVVISTLGNIKLQQDNPTGDRFAELKFTSSMVQSVGT   107
11-LDH               P-FA-HPVWVRAG--SYGDLEGARAVVLAAGVAQRPGE----TRLQLLDRNAQVFAQVVP   101
12-LDH               S-FYPTVSIDGSD--DPEICRDADMVVITAGPRQKPGQ----SRLELVGATVNILKAIMP   110
13-LDH               V-FAPKPVDIWHG--DYDDCRDADLVVICAGANQKPGE----TRLDLVDKNIAIFRSIVE   108
                                                     102       109

                     hhhh     ssss    hhhhhhhhhhhhh       sss hhhhhhhhhhhhhhh     s
1-MalDH              GIKEHAPDAFVICITNPLDAMVWALQKFSGLPTNKVVGMAGVLDSARFRHFLAEEFGVSV   165
2-MalDH              QAAPLSPNAVIIMVNNPLDAMTYLAAEVSGFPKERVIGQAGVLDAARYRTFIAMEAGVSV   164
3-MalDH              GIKKYAPEAFVICITNPLDAMVWALQKFSGLPAHKVVGMAGVLDSARFRYFLSEEFNVSV   165
4--intermediate      KIAEIC-DTKIFVITNPVDVMTYKALVDSKFERNQVFGLGTHLDSLRFKVAIAKFFGVHI   167
5--intermediate      GVAEENPEAIVLVVTNPVDVLTYVALKVSGLPKNRVIGLGTHLDSMRFKVLIAKHFNVHM   167
6--intermediate      KLRDRNPGALYVVVTNPVDVMTMVLDDVIGS-KGTVIGTGTSLDTFRFRAAVSELLNVPI   167
7--intermediate      KIVENAPESKILVVTNPMDVMTYIMWKESGKPRNEVFGMGNQLDSQRLKERLYNAG--AR   161
8--intermediate      EIVKRTKEAMIIMITNPLDVATYVVSTQFDYPRNLILGTGTMLETYRFRRILADKYQVDP   169
9--intermediate      SAMENGFNGFIVVVSNPVDIMTYVAYKVSGLPANQIIGSGTLLDSARLQCHIADCIDVDT   166
10-intermediate      NLKESGFHGVLVVISNPVDVITALFQHVTGFPAHKVIGTGTLLDTARMQRAVGEAFDLDP   167
11-LDH               RVLEAAPEAVLLVATNPVDVMTQVAYRLSGLPPGRVVGSGTILDTARFRALLAEYLRVAP   161
12-LDH               NLVKVAPNAIYMLITNPVDIATHVAQKLTGLPENQIFGSGTNLDSARLRFLIAQQTGVNV   170
13-LDH               SVMASGFQGLFLVATNPVDILTYATWKFSGLPHERVIGSGTILDTARFRFLLGEYFSVAP   168
                                   141                         168 171     181

                     S      ss        ss hhhh ss                  hhhhhhhhhhhhhhhh
1-MalDH              EDVTAFVLGGHGDDMVPLTRYSTVAGVPLTDLVKLGWTT---QEKLDAMVERTRKGGGEI   222
2-MalDH              EDVQAMLMGGHGDEMVPLPRFSTISGIPVSEFI-----A---PDRLAQIVERTRKGGGEI   216
3-MalDH              EDVTVFVLGGHGDSMVPLARYSTVAGIPLPDLVKMGWTS---QDKLDKIIQRTRDGGAEI   222
4--intermediate      DEVRTRIIGEHGDSMVPLLSATSIGGIPIQKFERFK------ELPIDEIIEDVKTKGEQI   221
5--intermediate      SEVHTRIIGEHGDTMVPVISSTSVGGIPVTRMPGWE------DFDVEEAVREVKEAGQRI   221
6--intermediate      VAVDGYVVGEHGEEAFVAWSTVTIKGIHIDQYIKERNI----NISREQIEKYVKDVAASI   223
7--intermediate      NIRRAWIIGEHGDSMFVAKSLADFDG----------------EVDWEAVENDVRFVAAEV   205
8--intermediate      KNINGYVLGEHGNAAFVAWSTTGCAGFPIDDLDEYFHRT--EKLSHEAVEQELVQVAYDV   227
9--intermediate      KSIHAYVLGEHGDSEMIPWSTVRVGGKGIYQIIKDNPTR-MNEGMFDSIHEAVKKDGWEI   225
10-intermediate      RSVSGYNLGEHGNSQFVAWSTVRVMGQPIVTLADAG------DIDLAAIEEEARKGGFTV   221
11-LDH               QSVHAYVLGEHGDSEVLVWSSAQVGGVPLLEFAEARGRA-LSPEDRARIDEGVRRAAYRI   220
12-LDH               KNVHAYIAGEHGDSEVPLWESATIGGVPMSDWTPLPGHDPLDADKREEIHQEVKNAAYKI   230
13-LDH               QNVHAYIIGEHGDTELPVWSQAYIGVMPIRKLVESKGEE-A-QKDLERIFVNVRDAAYQI   226
                                 195   199

                     hh  hhhsshhhhhhhhhhhhhhhhh     ssssssss hhh   sssssssssss  s
1-MalDH              VNLLKTGSAFYAPAASAIAMAESYLRDKKRVLPCAAYLDGQY-GIDGLYVGVPVVIGENG   281
2-MalDH              VNLLKTGSAYYAPAAATAQMVEAVLKDKKRVMPVAAYLTGQY-GLNDIYFGVPVILGAGG   275
3-MalDH              VGLLKTGSAFYAPAASAIQMAESYLKDKKRVLPVAAQLSGQY-GVKDMYVGVPTVIGANG   281
4--intermediate      IR--LKGGSEFGPAAAILNVVRCIVNNEKRLLTLSAYVDGEFDGIRDVCIGVPVKIGRDG   279
5--intermediate      IE--TWGGSQFGPAQAITNLVRTILQDERRVLTVSAYLDGEIDGIRDVCIGVPARLGREG   279
6--intermediate      IA--SQGATIWGPAATFQEIVVSHLANESKIIPISLP--QNIEGVGRVAVSVPTIIS--G   277
7--intermediate      IK--RKGATIFGPAVAIYRMVKAVVEDTGEIIPTSMILQGEY-GIENVAVGVPAKLGKNG   262
8--intermediate      IN--KKGFTNTGIAMAACRFIKSVLYDEHTILPCSAVLEGEY-GIKDVALSIPRMVCADG   284
9--intermediate      FN--RKGNTCYGIAASTTRIVRALMFNESVVLPVSTYLDGQY-GQDGVFTSVPAILDATG   282
10-intermediate      LN--GKGYTSYGIATSAIRIAKAVMADAHAELVVSNRRD-----DMGMYLSYPAIIGRDG   274
11-LDH               IE--GKGATYYGIGAGLARLVRAILTDEKGVYTVSAFTPEVE-GVLEVSLSLPRILGAGG   277
12-LDH               IN--GKGATNYAIGMSGVDIIEAVLHDTNRILPVSSMLKDFH-GISDICMSVPTLLNRQG   287
13-LDH               IE--KKGATYYGIAMGLARVTRAILHNENAILTVSAYLDGLY-GERDVYIGVPAVINRNG   283
                                246  250
```

Supplementary Figure S2. Sequence alignment of a subset of LDHs, MalDH type 3, and enzymes of the intermediate group. MalDH type 3 (R at position 102): (01) *Methylorubrum extorquens*, (02) *Chloroflexus aurantiacus*, (03) *Brucella abortus*. Enzymes of the intermediate group: functional as MalDH (R at position 102), (04) *Methanocaldococcus jannaschii*, (05) *Methanopyrus kandleri*, (06) *Ignicoccus islandicus*, (07) *Archaeoglobus fulgidus*, (8) *Selenomonas ruminantium*, (9) *Holdemanella biformis*: functionality not clearly defined (R and Q are absent at position 102), (10) *Weissella confusa*. LDH (Q at position 102): (11) *Thermus thermophilus*, (12) *Bifidobacterium longum*, (13) *Geobacillus stearothermophilus*. Normalized numbering in bold below key residues is according to Eventoff et al., 1977. Red arrows indicate positions 141, 181, and 250 used for distances measurements with R171 in MD simulations. They are highlighted in yellow in the *A. ful* MalDH sequence (7).

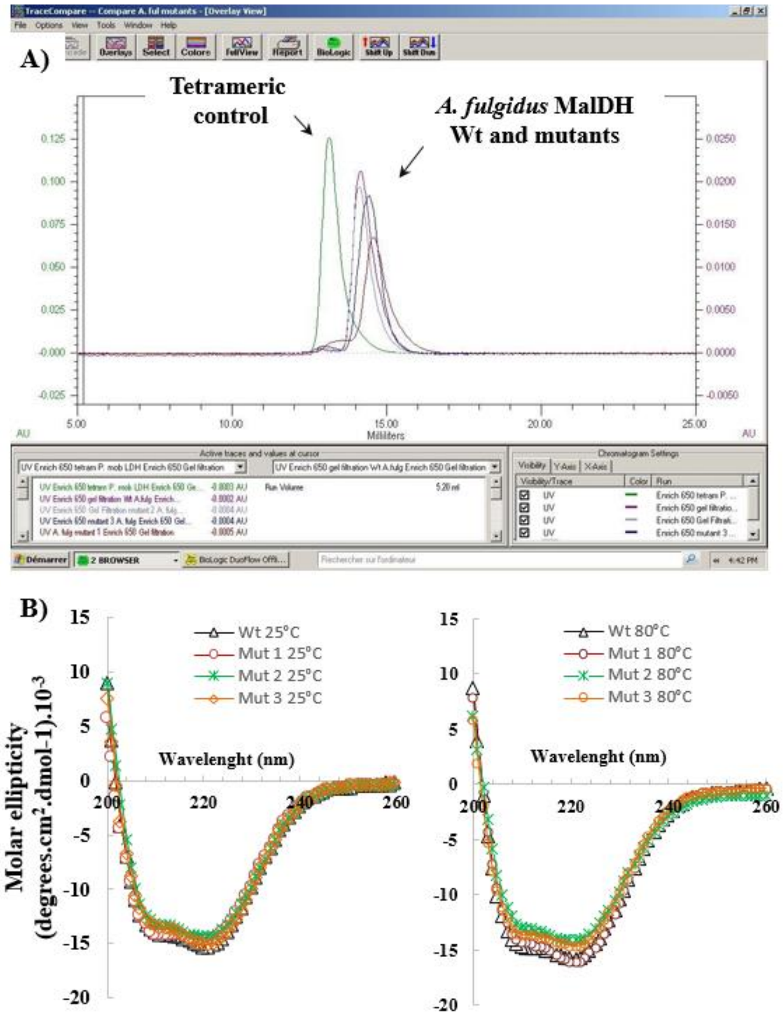

Supplementary Figure S3. (A) Enrich 650 SEC elution profiles of Wt *A.ful* MalDH and mutants. The tetrameric LDH from *Petrotoga mobilis* was used as control. The superimposition was done using the compare option of the Biologic software (Biorad). (B) Effect of thermal treatment on the conformational stability of Wt *A. ful* MalDH and mutants. The CD spectra were recorded at 25°C and after incubation at 80°C for 15 min.

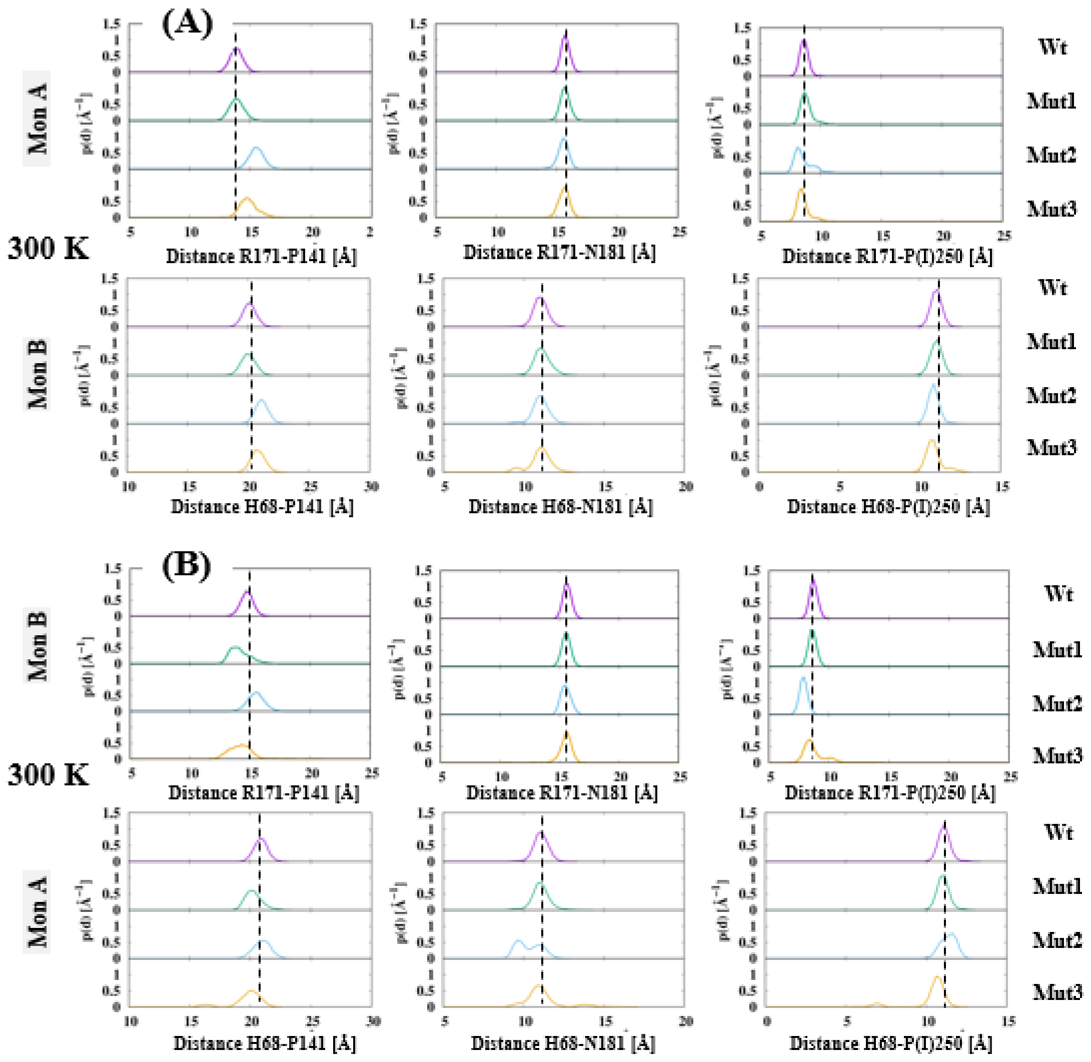

Supplementary Figure S4. Effect of mutations on the R171 and H68 conformational sub-state sampling at 300K. The figure shows the probability distributions of the distances between Cγ atoms of R171 and H68 and the Cα atoms of three different residues: P141, left; N181, central; P(I)250, right. The small inset shows the position of the various considered amino acids in the crystal structure. R171 is inside (in) the catalytic site. (A) Fluctuations of R171in monomer A and adjacent H68in monomer B. (B) Fluctuations of R171in monomer B and adjacent H68in monomer A. The dashed line refers to distance distribution for the Wt *A. ful* MalDH. The color code is purple, green, pale blue, and orange, for Wt *A. ful* MalDH, mutant 1, mutant 2, and mutant 3, respectively.

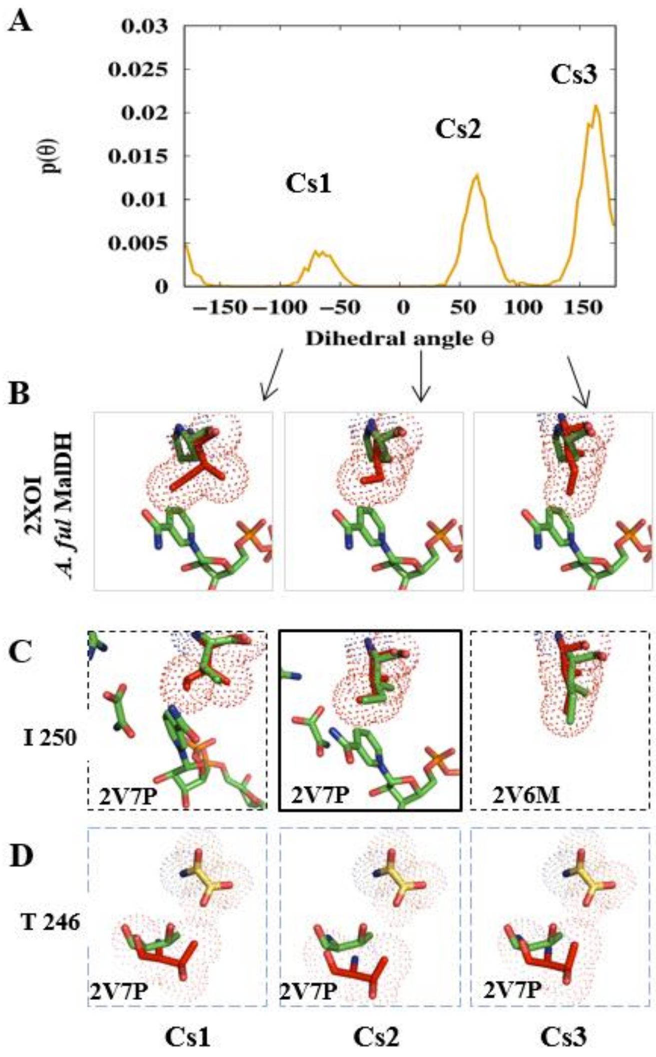

Supplementary Figure S5. Side chain fluctuations of I250 and T246 in mutant 3 at 340K. (*A*) Probability distribution functions of the dihedral angle defined by the atoms CA – CB – CG1 – CD of I250 on Chain A. (*B*) Panels show the conformational sub states of I250 (red sticks and dots) for representative values of the dihedral angle of I250 in Mutant 3. Named as Cs1, Cs2 and Cs3, respectively. Superposition between each Cs and the *A. ful* MalDH structure (2XOI). NADH and P250 are shown as green sticks. (*C*) Superposition of I250 in mutant 3 with *T. the* LDH structure in the T-state (2V6M) or R-state (2V7P). (*D*) Superposition of T246 in mutant 3 with *T. the* LDH structure in the R-state (2V7P) with red and green sticks, respectively. The substrate analog is in yellow sticks.

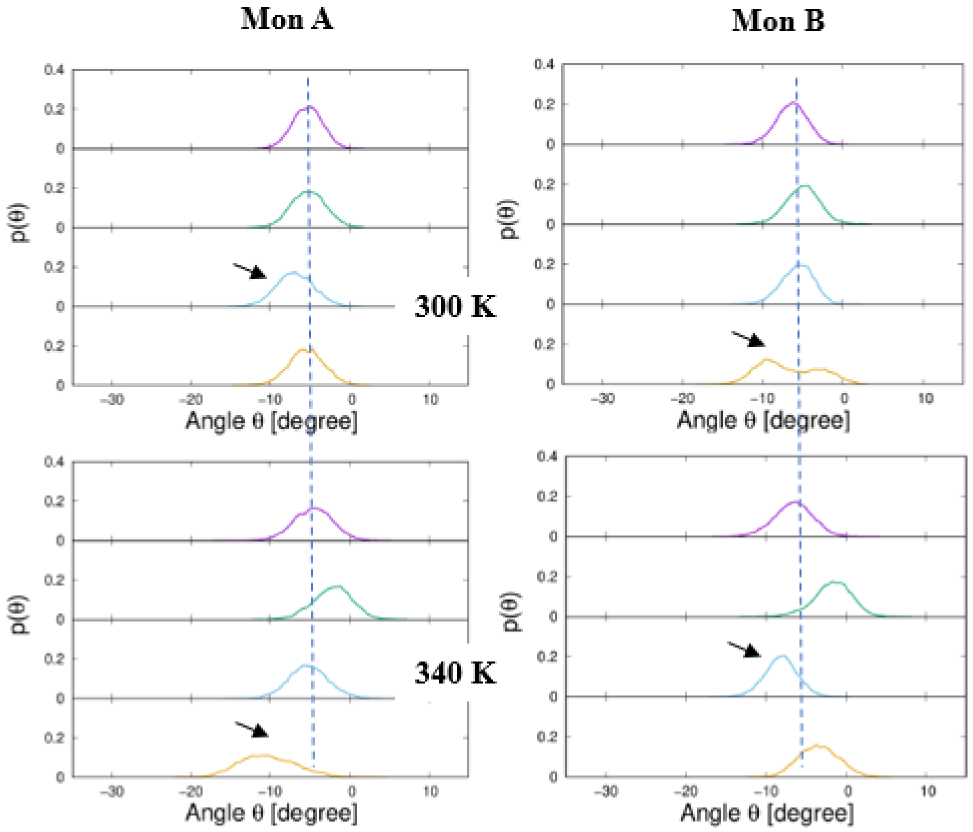

Supplementary Figure S6. The figure shows the probability distributions of the α2H angle for each monomer as indicated at two temperatures. The *A. ful* MaDH crystallographic structure is taken as reference. The color code is purple, green, pale blue, and orange, for Wt *A. ful* MalDH, mutant 1, mutant 2, and mutant 3, respectively. The arrows indicate noticeable decreasing values with respect to the most frequently sampled state (dashed lines).

In mutant 3, the angle helix variation may reaches values of 13 Å (monomer A at 340K). This value correspond to the one obtained from structural comparison between T and R states of an allosteric LDH (Iwata et al. 1994).

Iwata, S., Kamata, K., Yoshida, S. *et al.* T and R states in the crystals of bacterial L–lactate dehydrogenase reveal the mechanism for allosteric control. *Nat Struct Mol Biol* **1,** 176–185 (1994).

Linear numbering	Universal numbering
H47	H68
R81	R102
P120	P141
D147	D168
R150	R171
N157	N181
H172	H195
M179	M199
M179	M199
T212	T246
P216	P250

Table S1. The table shows the primary sequence numbering of important amino acids of *A. fulgidus* MalDH with their corresponding position on the normalized numbering system for LDH.
